# Structural and vibrational properties of agrellite

**DOI:** 10.1038/s41598-020-72631-1

**Published:** 2020-09-23

**Authors:** Ekaterina Kaneva, Alexandr Bogdanov, Roman Shendrik

**Affiliations:** Vinogradov Institute of Geochemistry, Siberian Branch of the Russian Academy of Sciences, Irkutsk, Russia 664033

**Keywords:** Theory and computation, Solid-state chemistry, Chemical physics

## Abstract

Agrellite, NaCa_2_Si_4_O_10_F, is a tubular silicate mineral which crystal structure is characterized by extended [Si_8_O_20_]^8–^ tubes and has a two-dimensional channel system. The mineral is a representative of a complex silicate family which contains some structural voids but cannot be considered as microporous because of small channel widths. However, the channel system of such minerals is able to host single guest atoms, molecules or radicals which can affect their physical properties. Presently, the exact mechanism of such hosting is undetermined. However, such information could be quite useful for materials’ application as zeolites as well as for a better understanding of their formation mechanisms. In this work we couple X-ray diffraction, infrared (IR) spectroscopy and ab initio calculations to identify structural features in agrellite from Malyy Murun massif (Russia) caused by incorporation of either H_2_O or OH^−^ into the channel system. We construct structural models of water-containing NaCa_2_Si_4_O_10_F and identified H_2_O positions. The derivation of H_2_O sites is based on simulation of IR-spectra. Infrared spectroscopy in combination with the ab initio calculation has proven to be an effective tool for the identification of the structural positions of hydroxyl anions (OH^−^) and neutral water groups (H_2_O) in minerals.

## Introduction

Recently, agrellite has received much attention as a luminescence material and has been studied extensively. It was discovered that materials based on agrellite, doped with a certain amount of rare earth ions, can be efficient white light emitter^1^ and promising phosphors^2^.

The chemical composition of agrellite is essentially NaCa_2_Si_4_O_10_F, although small amounts of different cations that substitute for calcium have been known since the first analyses of this mineral were made. In agrellite from Quebec (Canada) lanthanide ions (REEs) substitute Ca (up to 0.24 REE atoms per formula unit)^3,4^. References^5,6^ showed that calcium ions may partially be replaced by strontium in agrellite from Murun massif (Russia) (up to 0.46 Sr apfu). Recently we reported chemical data for agrellite samples from Dara-i-Pioz (Tajikistan) and Murun (Russia) massifs, pointing out that the REEs and Sr are present in both structures in minor amounts (≤ 0.04 apfu)^2^.

**Figure 1:**
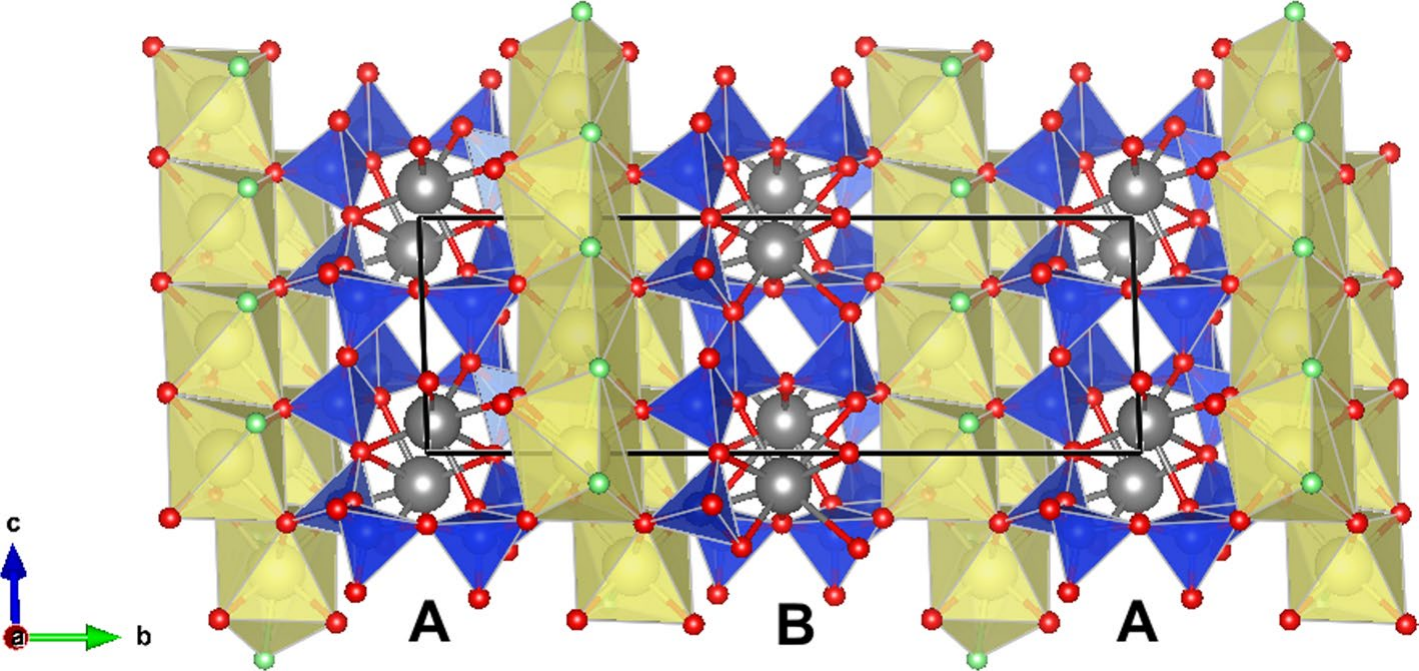
Perspective view of the crystal structure of agrellite projected down to *a* axis. SiO_4_ tetrahedra and Ca-polyhedra are drawn in blue and yellow, respectively; sodium, fluorine and oxygen atoms are drawn in grey, green and red, respectively. Unit cell edges and A- and B-tubes are designed.

According to the silicate minerals hierarchy of^7^, agrellite is a tube silicate with a one-dimensional tetrahedral polymerization. The [Si_8_O_20_]^8−^ -tube in agrellite extends along the *c*-axis and consists of two linked chains of four-membered rings, each topologically identical to the chain in vlasovite structure (see^8, 9^). The silicon-oxygen radical has the designation ^3^T8, where T means “tetrahedron”, 3 is the connectivity of the tetrahedron and 8 is the number of such tetrahedra in the geometrical repeat unit^7^. Adjoining four-membered rings of opposing chains are interconnected across the tube by two tetrahedra, forming eight-membered rings that link along the *a*-axis and a six-membered ring viewed along the *c*-axis. The ^3^T8 tubes are connected with bands of (CaO_5_F_2_)^12–^ polyhedra and (CaO_5_F)^12–^ octahedra. Na-polyhedra occupy voids inside the tubes (Fig. 1).

The same type of tube (^3^T8) can be found in the crystal structures of minerals of litidionite group, which includes litidionite^10^, fenaksite^11^, manaksite^12^ and calcinaksite^13^, and related synthetic compounds (Na_2_Cu[Si_4_O_10_]^14^, Na_2_Co[Si_4_O_10_]^15^, Na_2_Ni[Si_4_O_10_]^15^, KNaCu[Si_4_O_10_]^16^, KNaFe[Si_4_O_10_]^16^, KNaMn[Si_4_O_10_]^16^, Na_2_Mn[Si_4_O_10_]^17^, K_2_Ca[Si_4_O_10_]^18^ and K(K,Na)Mn[Si_4_O_10_]^19^. However, agrellite is not isostructural with these phases. In litidionite, KNaCu[Si_4_O_10_], tetrahedral tubes, extended parallel to the *a*-axis, link along the *c*-axis via bands of Cu- and Na-polyhedra, and the pores within the tube are occupied by K ions. Fenaksite and manaksite are the Fe^2+^- and Mn^2+^-analogues of litidionite. Calcinaksite is the Ca^2+^-analogue of litidionite that also contains H_2_O. The presence of water molecules in calcinaksite was confirmed by single-crystal X-ray diffraction analysis^13^, as well as using IR spectroscopy^20^: there are absorption bands at about 3540, 3340 and 3170 cm^−1^ corresponding to the O–H-stretching vibrations of H_2_O molecules, and peak at 1654 cm^−1^, assigned to the bending vibration of H_2_O molecules. At present, calcinaksite is the only water-containing representative of this structural group.

It is known that small amounts of water molecules can be incorporated into nominally anhydrous minerals. Manaksite and fenaksite have very weak bands in their IR spectra, observed at 3621 and 3615 cm^−1^, respectively, indicating trace amounts of H_2_O molecule^20^.

Early we reported the presence of minor water content in agrellite, discovered utilizing IR spectroscopic studies^2^.The vibrational spectrum contains a wide band in the 2800–3700 cm^−1^ spectral region, which corresponds to the symmetric and asymmetric stretching vibration of the H_2_O. No indications for hydroxyl anions and no clear maxima attributable to H_2_O groups have been found during the structural study^2^, which means the localization of the molecules remains controversial. Whereas H_2_O can be identified by IR spectroscopy, it is not clear how the molecules are incorporated into the structure of agrellite. However, from infrared spectroscopic studies, it now seems clear that there are several sites that H_2_O can occupy in agrellite structure. In addition, the changes in the crystal structure due to the incorporation of water molecules are of important relevance.

The agrellite structure provides an excellent template within which to study the possibility of IR spectra modeling and prediction of the appearing of IR peaks corresponding to Si–O–Si, O–H vibrations of H_2_O molecules and hydroxyl groups in natural and synthetic silicates.

In the present article, a multi-method approach is applied to study the crystal structure of agrellite from the Murun massif (Russia), including an accurate crystal-chemical characterization, ab initio calculations and IR spectroscopy. Using the techniques it has become possible to provide distortion characteristics of the mineral structure and theoretically calculated structural models of water-containing phases of agrellite.

## Results

### Structure description and crystal chemistry

The projection of crystal structure parallel to the *a*-axis is shown in Fig. 1. Ghose and Wan^4^, carrying out a crystal structure determination on the Canadian agrellite, proposed to designate two crystallographically distinct silicate tubes and its atoms as A and B. Thus, the same symbols were adopted in this paper for the convenience of comparison between the corresponding structural units. Figures showing structural details were prepared using the program VESTA (version 4.3.3)^21^.

The average composition (determined over nine spots) and the atom proportions in atoms per formula units (apfu) derived on the basis of four Si cations are reported in Supplementary Table S1. OH content was calculated using a single-crystal X-ray diffraction data refinement (occupancies of F(1A) and F(1B) sites) based on the lack of fluorine content (Supplementary Table S1). The H_2_O weight percentage was derived from calculation assuming “Total wt%” = 100%.

The composition of the studied agrellite is almost identical to that of the other Murun samples reported by^2^ except for the lower CaO (26.6(3) vs. 27.1 wt%, respectively) and F content (3.6(3) *vs.* 4.5 wt%, respectively).

Unit cell parameters, relevant data of the X-ray collection and the structure refinements are given in Supplementary Table S2, whereas final atomic coordinates, site occupancies, equivalent/isotropic and anisotropic displacement parameters are reported in Supplementary Tables S3 and S4. Relevant cation–anion bond lengths for Si-tetrahedra and Ca- and Na-polyhedra are given in Supplementary Tables S5 and S6.

One of the aims of this study was to give a theoretical account of the effect of water molecules incorporation in the structure of agrellite. The degree of distortion of the coordination polyhedra was calculated to test the geometrical flexibility of the agrellite crystal structure and hypothetical water-containing analogues (see “Methods” section). The distortion parameters are represented in Supplementary Tables S5, S6.

#### Cation sites

In the agrellite crystal structure, the symmetrically independent crystallographic cation sites are: eight tetrahedrally coordinated Si; two octahedrally coordinated Ca (Ca(1B) and Ca(2A)), two [8]-coordinated Ca (Ca(1A) and Ca(2B)) and two [8]-coordinated Na sites.

Tetrahedra distances are quite similar in both A and B tubes: the measured Si–O individual distances range from 1.564(3) to 1.654(3) Å. All tetrahedra evidence a notable shortening of unshared Si–O bond lengths (< Si-O_unsh_ >  ~ 1.578 Å) concerning shared ones (< Si-O_sh_ >  ~ 1.634 Å) (Supplementary Table S5). This feature is pronounced in the crystal structure of vlasovite^9^ as well as in structurally and chemically related minerals from Murun massif (for instance, miserite^22^, frankamenite^23^, tinaksite and tokkoite^24^). Generally, short Si–O bands are associated with large O–Si–O angles^25^. O–Si–O associated with unshared Si–O bonds is ~ 113° instead of the ideal value of 109.47°.

Generally, all distortion parameters of symmetrically relative tetrahedra of both tubes are very similar. Note that the crystal structure exhibits the tetrahedra sites lightly distorted (Supplementary Table S5). The main differences between the tetrahedra distortion parameters involve the TAV parameter that ranges from about 5.5 to ~ 37.7.

Relevant information about coordination number, cation site population, mean atomic number and average cation–anion bond length for polyhedra are given in Table 1. A satisfactory agreement between mean electron numbers and average interatomic distances as derived by X-ray and EPMA measurements was found. The interatomic distances have been calculated using the Shannon ionic radii^26^.

**Table 1:** Polyhedral cation distribution, coordination number (CN) and mean atomic numbers (m.a.n., e^−^) of cation sites, polyhedral mean distances (Å), as determined by structure refinement (X-ray) and chemical analysis (EPMA).

Site		CN	e^−^_X-ray_	e^−^_EPMA_	〈cation–anion〉_X-ray_	〈cation–anion〉_EPMA_
Ca(1A)	0.922Ca^2+^, 0.032Sr^2+^, 0.010LREE^3+^, 0.009Na^+^, 0.006Mn^2+^, 0.006Fe^2+^, 0.005Mg^2+^, 0.004 K^+^, 0.002Zr^4+^, 0.001Cu^2+^	8	20.500	20.90	2.538(9)	2.44
Ca(1B)	0.986Ca^2+^, 0.012HREE^3+^	6	20.420	20.53	2.342(8)	2.33
Ca(2A)	0.966Ca^2+^, 0.004HREE^3+^	6	20.100	20.18	2.347(8)	2.34
Ca(2B)	0.902Ca^2+^, 0.052Sr^2+^, 0.010LREE^3+^, 0.009Na^+^, 0.006Mn^2+^, 0.006Fe^2+^, 0.005Mg^2+^, 0.004Ba^2+^, 0.002Zr^4+^, 0.001Cu^2+^	8	20.820	21.41	2.567(9)	2.47
Na(A)	0.942Na^+^, 0.046 K^+^	8	11.231	11.24	2.677(9)	2.54
Na(B)	Na^+^	8	11.011	11.00	2.599(9)	2.54

The crystal chemistry of the Ca-polyhedra is slightly different. Specifically, the mean atomic number (m.a.n.) varies from about 20 to 21 e^−^. This difference is justified by the different chemical content in Ca-octahedra and Ca-polyhedra. Ca(1B) and Ca(2A) are mainly occupied by Ca and minor heavy rare earth elements (HREE)—Er and Yb. The octahedra exhibit low values of mean atomic number (20.10 and 20.42 e^-^) and 〈Ca–O,F〉 distances (~ 2.342 and 2.347 Å vs. the ideal value of 2.35 Å) in relation to polyhedra. The most suitable host cations are Er and Yb as 〈HREE–O,F〉 distances are smaller than 〈Ca–O,F〉 ones (2.25 and 2.23 Å). The Ca(1A) and Ca(2B) exhibit greater mean atomic numbers (20.50 and 20.82 e^−^). It is reasonable that heavy cations replacing Ca preferentially occupy the polyhedral Ca sites. Recent EPR and luminescence studies carried out by us^2^ confirm that minor amounts of Sr, Mn, Fe, Ce and Mg in the Murun samples of agrellite are located in [8]-coordinated Ca(1A) and Ca(2B) positions. Table 1 shows the elements assigned to these positions. At the same time, the 〈Ca-O,F〉 distances are 2.538 and 2.567 Å that is somewhat higher with respect to the ideal for [8]-coordinated Ca value of 2.465 Å. These increased values are most likely due to the geometry of adjacent tetrahedral chains rather than to the content of the cationic position.

The bond-valence sum (BVS^27, 28^) is satisfactory for octahedrally coordinated Ca(1B) and Ca(2A) cations. The sums of the bond valence at the Ca(1A) and Ca(2B) sites are significantly lower than the expected ideal value (2.00 valence units (vu)) for full Ca site occupancy.

A comparison of the distortion parameters shows substantial similarities between the two Ca-polyhedra (Ca(1A) and Ca(2B)) (Supplementary Fig. S1). However, note that Ca(1B) octahedron, with respect to Ca(2A) one, has stronger distortion: (1) greater OAV value (43.214 vs. 28.501, respectively, see Supplementary Table S6); and (2) greater ELD (3.295 vs. 4.250, respectively, see Supplementary Table S6, Supplementary Fig. S1), the difference is 1.3 times.

Na is located at the Na(A) and Na(B) sites, within the eight-member rings of the silicate radical. Na(B) site (m.a.n. ~ 11.01 e^−^) is fully occupied by Na^+^. Na(A) site (m.a.n. − 11.231 e^−^), which nominally contains only Na atoms, has to be partially populated by cations with higher mean atomic number, for instance, K^+^. The bond valence sums for the atoms at Na positions are slightly lower than 1. Specifically, the sum of the bond valence at the Na(A) site is 0.91 vu, close to that (0.96 vu) calculated for Na(B) position. Supplementary Table S6 demonstrates that the values of BLD, ELD and volumes of the independent Na(A)- and Na(B)-polyhedra are quite similar.

#### Anion sites

In the agrellite crystal structure there are twenty independent oxygen atoms: 1(A) – 10(A) and 1(B) – 10(B); and two F positions. O4, O5, O8 and O10 (both A and B) are shared by a Si tetrahedron and Ca-polyhedra, and also, in some cases, enter the coordination polyhedron of Na located in an 8-membered tetrahedral ring. O1, O2, O3, O6, O7, O9 are shared by two Si tetrahedra and coordinate Na. Analysis of Supplementary Table S7 reveals that the bond-valence sums of the studied sample is generally satisfactory for the oxygen atoms. Some of them – the bridging oxygen atom positions between the SiO_4_-tetrahedra and Ca-polyhedra – are slightly undersaturated (1.81–1.90 vu). The shortening of Si–O distance and bridging anion valence deficiency of each tetrahedron is due to the different Si and Ca-site cations contributions to the valence saturation in the oxygen atoms. 2.20 and 2.22 vu values of O(6A) and O(6B) atoms indicate that these atoms are held strongly by the surrounded cations (i.e. those in the Si2, Si4 and two Na sites). Supplementary Fig. S2 represents the BVS, CN and 〈O-cation〉 values for each oxygen site in the crystal structure.

The calculated atomic proportion of F for the studied agrellite is equal to 0.76 apfu. Hydroxyl can be easily incorporated in the structures as a substitute for fluorine at its anion site. Based on the refinement of position occupancies (Supplementary Table S3), both F(A) and F(B) positions are filled. Thus, the calculated hydroxyl group content is 0.24 apfu. F(A) and F(B) are shared by three different Ca-polyhedra, and BVS values for these sites are slightly lower than 1 (i.e. 0.81 and 0.79 vu, respectively). A similar feature was recently pronounced in the crystal structure of fluorcarletonite^29^, a chemically related mineral from the Murun massif.

As in the previous work^2^, here it was not possible to find the positions occupied by water molecules. Based on the results of a chemical investigation, it is possible to calculate how much water can be contained in the formula unit of the mineral (Supplementary Table S1).

#### Crystal chemical formula

Considering the above results, the following crystal-chemical formula can be proposed for the studied agrellite from Murun massif (calculated on the basis of 4 Si apfu): (Na_0.971_K_0.024_)(Ca_0.912_Sr_0.042_REE_0.010_Na_0.009_Mn_0.006_Fe_0.006_Mg_0.005_K_0.002_Ba_0.002_Zr_0.002_Cu_0.001_)(Ca_0.976_REE_0.08_)[Si_4_O_10_](F_0.760_OH_0.240_)·0.087H_2_O.

#### Structural channels

**Figure 2:**
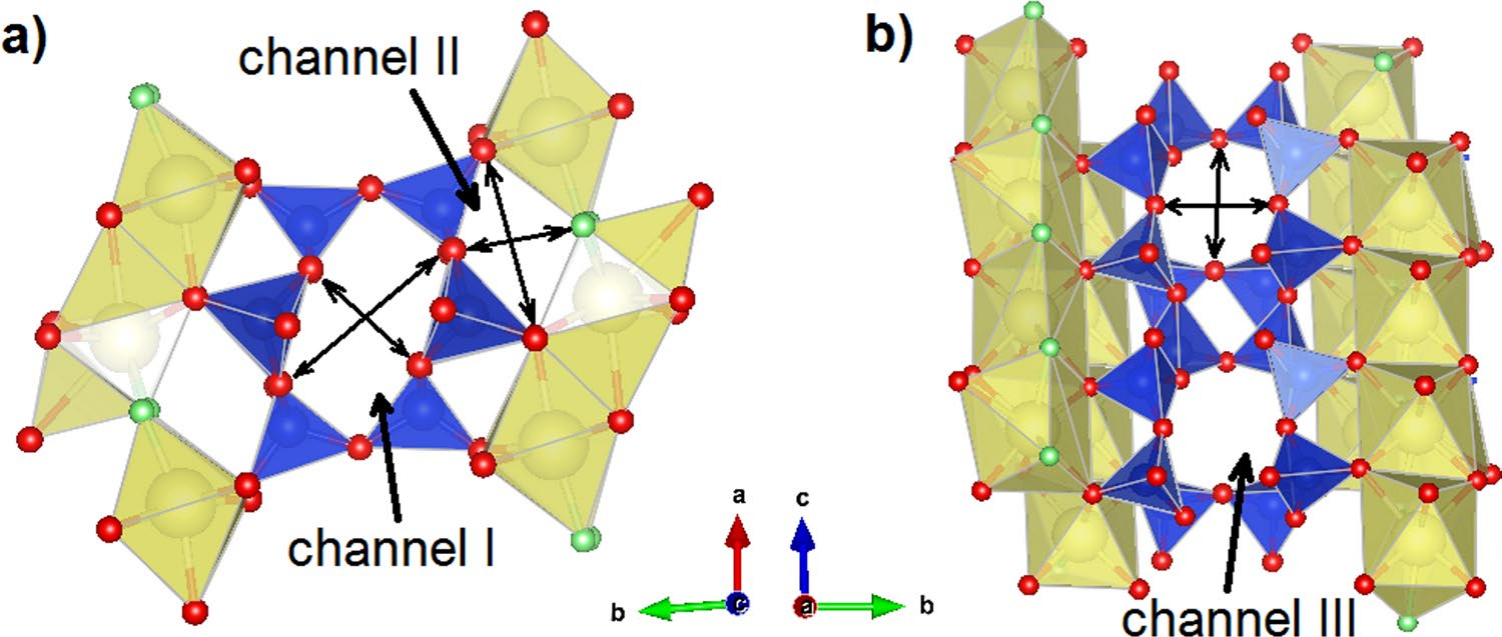
Perspective view of the agrellite crystal structure fragments projected down to *c* axis with apertures of channel I and II (**a**) and down to *a* axis with an aperture of channel III (**b**). For clarity, Na atoms in the channels I and III have been omitted.The shortest distances between oppositely located oxygen atoms in the ring are shown.

Three types of channels are distinguished inside the crystal structure of agrellite (Fig. 2). Channel I is extended along the *c*-axis and delimited by six-membered rings of tetrahedra (Fig. 2a). Shortest distances between oppositely located oxygen atoms in the ring are 5.161(4) × 3.083(4) Å for A-tube and 4.823(4) × 3.251(4) Å for B-tube. Channel II is formed by two tetrahedral chains and two Ca-polyhedral bands and extends parallel to the *c*-axis (Fig. 2a). The dimensions of the smallest free aperture of the channel are 4.821(4) × 3.374(4) Å and 4.638(4) × 3.028(4) Å for A- and B-tube, respectively. Channel III is delimited by eight-membered tetrahedral rings along the *a*-axis (Fig. 2b). The ring cross-section has free diameters of 6.983(5) × 3.573(4) Å (A-tube) and 6.983(5) × 3.464(5) Å (B-tube). Channel I and channel III intersect forming a 2-dimensional channel system. Sodium atoms are localized in common voids of two intersecting channels.

A fundamental characteristic of a channel, described the accessibility of the pore system to guest species, is *effective channel width* (*ecw*), that is defined as the distance between oxygen atoms in the smallest *n*-ring or smallest free aperture subtracted by 2.7 Å, when the oxygen ionic radius is assumed to be 1.35 Å^30^. In particular, the structure studied have the following values of effective channel dimension: channel I—2.5 × 0.4 and 2.1 × 0.6 Å, channel II—2.1 × 0.7 and 1.9 × 0.3 Å, channel III—4.3 × 0.9 and 4.3 × 0.8 Å for A- and B-tube, respectively (Table 2). According to^17^ a minimum *ecw* of 3.2 Å is required for a crystalline substance to be defined as microporous. In this sense, despite the channels occurring, agrellite cannot be considered as microporous. However, the pores inside the channels of agrellite have larger dimensions with respect to the channel aperture, and therefore theoretically may contain guest atoms, for instance, water molecules.Thus, in^2^, it was formulated inaccurately that water molecules do not have a specific structural position in the voids of the silicate tubes.

**Table 2:** Effective channel aperture dimensions (Å) in the agrellite and simulated H_2_O-containing agrellite models.

Channel	Tube	Agrellite	Model C1	Model C2	Model C3	Model C4
I	*A*	2.5 × 0.4	2.4 × 0.3	1.6 × 0.1	1.6 × 0.2	2.1 × 0.0
	*B*	2.1 × 0.6	2.1 × 0.4	1.8 × 0.4	1.6 × 0.5	**1.6 × 0.3**
II	*A*	2.1 × 0.7	2.1 × 0.5	2.3 × 0.3	2.2 × 0.5	2.0 × 0.2
	*B*	1.9 × 0.3	**2.1 × 0.3**	**2.1 × 0.0**	**2.1 × 0.0**	**0.7 × 0.0**
III	*A*	4.3 × 0.9	4.3 × 0.8	**4.3 × 0.9**	**4.3 × 0.8**	**4.3 × 0.8**
	*B*	4.3 × 0.8	4.3 × 0.6	**4.3 × 0.9**	**4.3 × 1.2**	**4.3 × 1.4**

The channels, in which the water molecules are located, are indicated in bold.

### Simulation of the crystal structure and IR spectra

The ab initio calculation of the infrared spectra of agrellite was carried out and compared with the experimental one.

**Figure 3:**
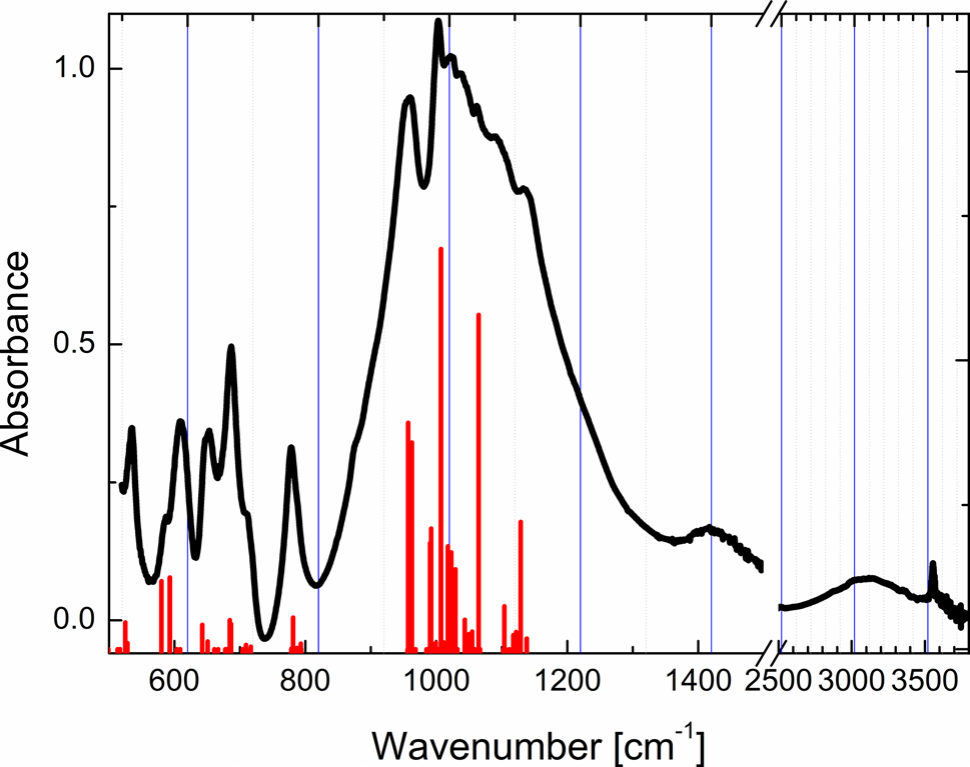
Calculated infrared spectra of agrellite: (**a**) initial structure, (**b**) agrellite with OH substituting fluorine, (**c**–**e**) agrellite containing one, three, four and five H_2_O molecules per unit cell. The area near 1500 cm^−1^ (marked ‘I’) accumulates bending modes of H_2_O, the area ‘II’—from 2200 to 3800 cm^−1^—contains symmetric and asymmetric stretching modes (see Table 4).

The peaks in the 500–1350 cm^−1^ region of the IR absorption spectrum (Fig. 3) are attributed to different types of Si–O–Si vibrations. The calculated and experimental values of the peak positions agree well and their assignments are given in Supplementary Table S8.

At 2900–3700 cm^−1^ wide band is observed. It is caused by H–O–H vibrations. The complicated shape of the band is due to differences in the environment of the H_2_O molecule positions in the agrellite crystal structure.

**Figure 4:**
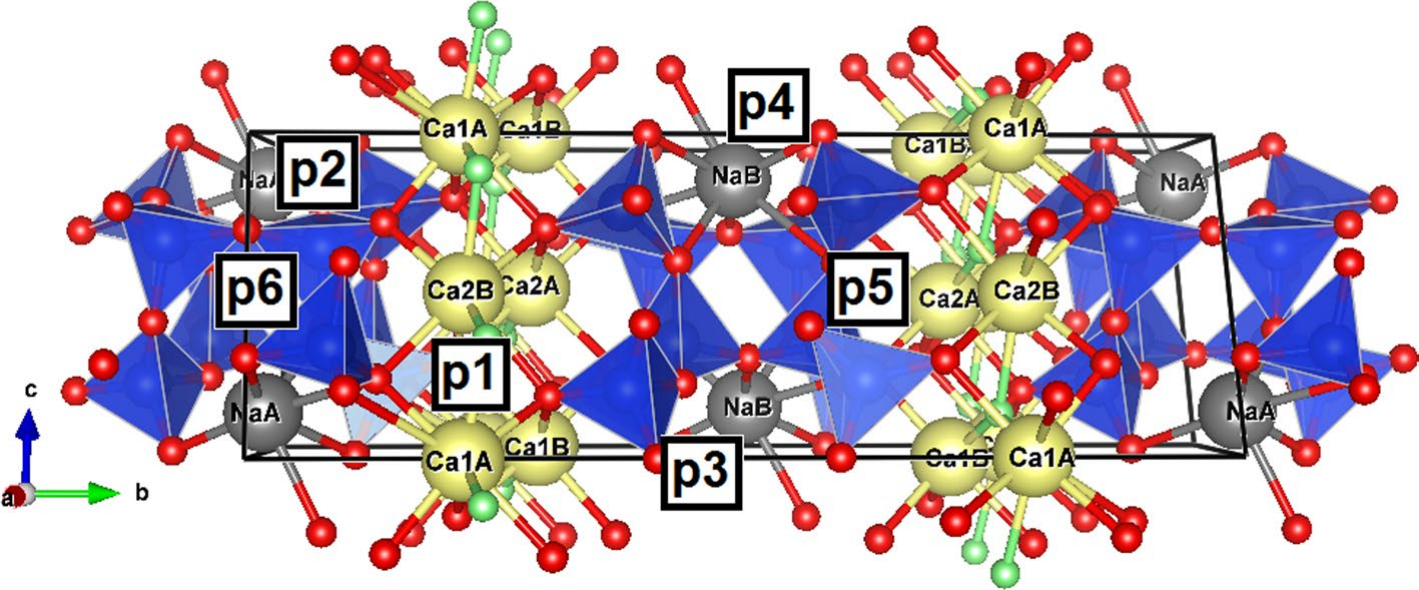
Schematic representation of H_2_O positions (p1–p6) distribution within simulation unit cells. Ca-polyhedra are not shown. The same digits enumerate corresponding water molecules (W), situated in these positions.

As a part of the simulation, the water molecules were placed within every suitable void in the agrellite crystal structure, from one to five water molecules per unit cell. The largest simulated water concentration in agrellite is 5.3 wt%, which is much greater than the experimentally determined value (~ 0.4 wt%, Supplementary Table S1). It was impossible to simulate even larger contents of water because the structure destroyed during optimization. We assume, however, this is sufficient to cover all possible absorption bands in the IR-spectrum. Figure 4 presents simulation unit cells with 1.1 to 5.3 H_2_O wt% with enumerated positions (p1–p6) of water molecules, located in the voids with a relatively large volume.

The exact way of incorporation of water molecules into the agrellite crystal structure is under discussion; however, one can make trivial energetic considerations by comparing total energies of the crystal structure with a single water molecule in different positions. Table 3 represents such total energies of agrellite with a single H_2_O molecule. The lowest energy is registered for the position p1 in Fig. 4, that is situated in channel II of B-tube between the tetrahedral chain and Ca-polyhedral bands. It can be assumed that the incorporation of an H_2_O molecule into the agrellite crystal structure at this position would have the greatest probability. Then, positions p2, p3, p4 and p5 have significantly higher energies. We assume these sites have less ability to host H_2_O. Finally, p6 appears the least favorable one for H_2_O hosting. Further in the text, the enumeration of the water molecules (W1–W6) will be consistent with the positions (p1–p6) in Fig. 4.

**Table 3:** Relative energies of the agrellite crystal structure with a single H_2_O molecule at the different positions.

Position	p1	p2	p3	p4	p5	p6
Energy, eV	0.00	+ 0.82	+ 0.97	+ 1.08	+ 1.10	+ 2.11

**Figure 5:**
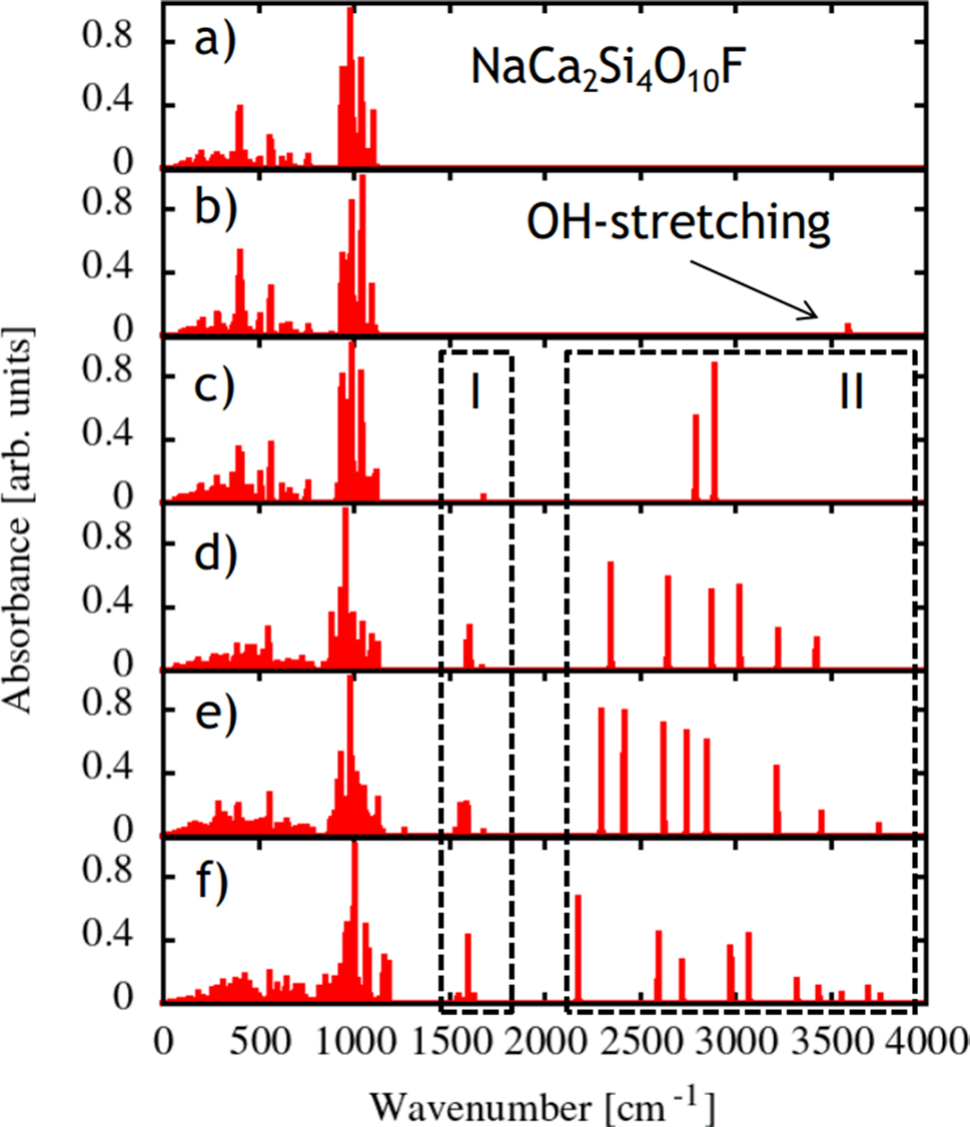
The experimental absorption spectrum and calculated IR-absorption peaks in agrellite. Calculated peaks are shifted by about 20 cm^−1^ to low energy region in comparison with the experimental scale.

The simulated IR spectra are shown in Fig. 5. The main absorption bands of agrellite are located in the region from 200 to 800 cm^−1^ and from 900 to 1150 cm^−1^.

Introduction of OH group substituting F atom produces a peak at about 3611 cm^−1^ associated with OH^−^ stretching vibrations and has only a slight effect on the other part of the IR spectrum.

The changes caused by adding water molecules affect two parts of spectra. H_2_O bending vibrations appear in the 1500–1700 cm^−1^ spectral region. The symmetric and asymmetric stretching vibrations of H_2_O appear in the region of 800–2200 cm^−1^ depending on the water position in the crystal structure. In general, asymmetric vibrations have lower frequencies than symmetric ones; however, this is not a rule in agrellite structure. The calculated frequencies of H_2_O in agrellite are collected in Table 4 with vibration types assigned.

**Table 4:** Energies (cm^−1^) and type of vibrations of H_2_O molecules at different positions (p1–p6) in the structural models (C1–C4) of agrellite.

Model	Vibration	p	Energy, cm^−1^	Model	Vibration	p	Energy, cm^−1^	Model	Vibration	p	Energy, cm^−1^
C1	B	1	1680	C3	B	3	1537	C4	B	4	1549
C1	AS	1	2794	C3	B	4	1560	C4	B	1	1582
C1	SS	1	2893	C3	B	2	1597	C4	B	2	1601
				C3	B	1	1683	C4	B	5	1616
C2	B	3	1595	C3	AS	4	2300	C4	B	6	1635
C2	B	2	1612	C3	AS	2	2419	C4	AS	2	2178
C2	B	1	1676	C3	AS	1	2629	C4	AS	6	2599
C2	AS	2	2351	C3	SS	3	2747	C4	SS	1	2725
C2	AS	1	2648	C3	SS	1	2853	C4	SS	5	2979
C2	SS	1	2880	C3	SS	4	3222	C4	SS	4	3074
C2	AS	3	3027	C3	SS	2	3454	C4	AS	1	3327
C2	SS	3	3226	C3	AS	3	3758	C4	SS	2	3442
C2	SS	2	3427					C4	SS	6	3559
								C4	AS	5	3699
								C4	AS	4	3763

*SS* symmetric stretching, *AS* asymmetric stretching, *B* bending.

The simulated crystal structures need to be examined in the space group *P*1 since the entry of water molecules does not have a symmetrical principle and affects the structural units by different distortion mechanisms. The number of atoms in the asymmetric unit of the structure when described in sp. gr. *P*1 is doubled due to the lower symmetry. Therefore, the atom labels are assigned additional symbols “1” and “2” (e.g. Si(1A)1 and Si(1A)2 and so on). Atomic coordinates of simulated models C1, C2, C3 and C4 are reported in Supplementary Tables S9–S12, respectively.

#### Agrellite structural model with one OH-group in the unit cell (NaCa_2_Si_4_O_10_(F_0.75_OH_0.25_))

**Figure 6:**
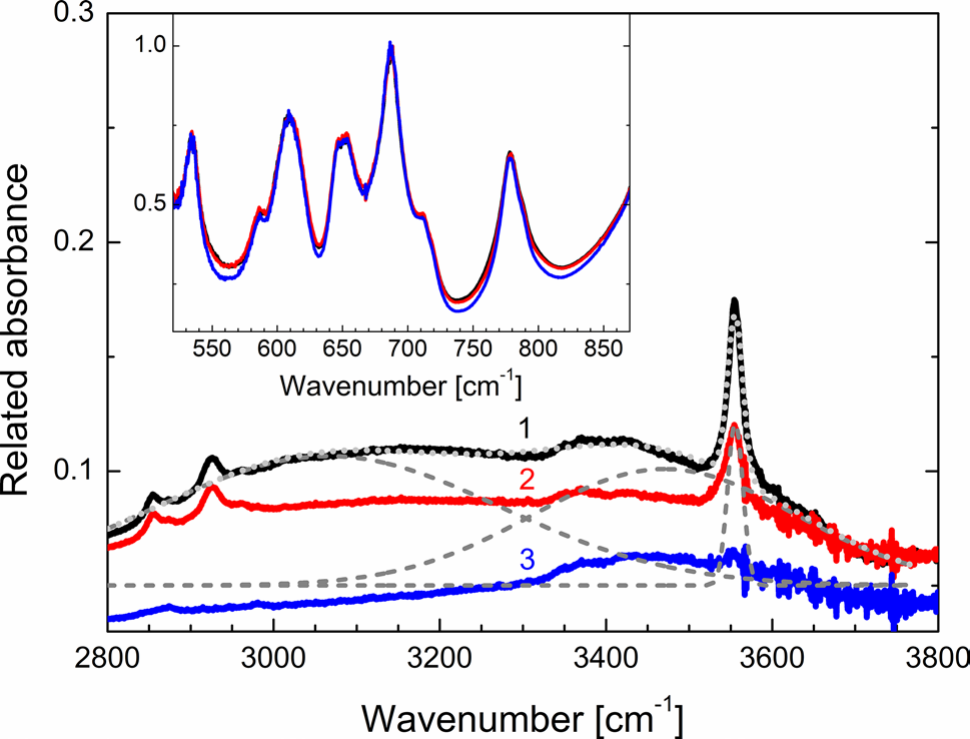
Absorption spectra of the initial (black curve 1) and preheated up to 280 °C (red curve 2) and 480 °C (blue curve 3) agrellite samples. Dashed gray curves are Gaussian deconvolution of the initial agrellite absorption spectrum in region 2800–3700 cm^−1^, gray dotted curve is their sum. The region 520–870 cm^−1^ is enlarged in the inset.

The substitution of fluorine atoms for OH-groups does not imply significant structural changes since the ionic radii of the fluorine and oxygen are very close (1.30 and 1.36 Å, respectively^26^), and the hydrogen ion does not have a strong effect on its environment. Figure 5b shows the simulated IR spectrum of agrellite with OH at F(A) position on which a single peak at 3611 cm^−1^ corresponds to OH stretching vibration. This peak is located within the range 2800–3700 cm^−1^ where IR absorption is observed experimentally (Fig. 6). Therefore OH is definitely present in the agrellite crystal structure as fluorine substitute.

The single OH group placed in other fluorine position F(B) produces very similar IR spectra. The splitting of OH vibration frequency between F(A) and F(B) positions is only 14 cm^−1^, while host bands remain almost unchanged which confirms the only slight effect of OH group on the crystal structure.

#### Agrellite structural model with one H_2_O molecule in the unit cell (NaCa_2_Si_4_O_10_F·0.25H_2_O): model C1

The only water molecule (W) in the unit cell of this structure is located in channel II of B-tube between the tetrahedral chain and Ca-polyhedral bands (position p1 in Fig. 4). O_W1_–H1 and O_W1_–H2 distances are ~ 1.02 Å (H1–O_W1_–H2 angle is 105.5º). The oxygen atom of the water molecule is coordinated by Ca(1A)2 with a bond length of ~ 2.33 Å (Supplementary Fig. S3). The shortest H1···O distance is 1.51 Å, whereas H2 is distant from F(B)2 atom by 1.41 Å. In addition, Si(2B)1 and Si(4B)1 atoms are situated at a distance of 2.55 and 2.85 Å, respectively, from the H_2_O molecule.

Thus, a major expectable consequence of the water molecule incorporation is that it affects the linkages within the Ca-polyhedra band and Na atoms. Indeed, in this crystal structure, the Ca(1A)2 changes its coordination number from 8 to 9, since the oxygen of the water molecule becomes one of the vertices of its polyhedron. Na atoms, except 8-coordinated Na(B)1, occur in seven-fold coordination.

BVS calculations were also used to provide insight into the valence state of the cations in the agrellite models (Supplementary Tables S13 and S14).Tetrahedral distances of A- and B-tube silicate radicals are slightly longer than in natural agrellite crystal structure (average distances Si–O = 1.63–1.64 Å *vs* 1.62 Å, see Supplementary Tables S5 and S13). The BVS values are also similar (~ 3.8–3.9 vu, Supplementary Tables S5 and S13). The Si(2B)1 tetrahedron displays the highest TAV (77.13) and TQE (1.019) parameters. The Si(4B)1 specifically, and all other tetrahedral are less distorted by the entry of the H_2_O molecule. The BLD parameter for all tetrahedra does not exceed 1.65%.

The dimensions of the smallest free aperture of channels and the values of effective channel dimensions do not differ strikingly from those in the structure of natural agrellite (Table 2).

Summarizing all the data above, a water molecule in this modeled structure is coordinated by the calcium cation and strongly fixed in the cavity of the channel, unable to migrate along it, and does not have a strong distorting effect on the internal structure of agrellite.

The calculated values of symmetric stretching, asymmetric stretching and bending vibrational modes of the W1 molecule in position p1 of the structural model C1 are given in Table 4. The theoretical IR spectrum is shown in Fig. 5c.

#### Agrellite structural model with three H_2_O molecules in the unit cell (NaCa_2_Si_4_O_10_F·0.75H_2_O): model C2

The modeling of the crystal structure yielded W1 water position, (O_W1_ is bonded to H1 and H2 with distances of 1.03 and 1.02 Å, H1–O_W1_–H2 angle is 104.5º), W2 (O_W2_ is bonded to H3 and H4 with distances of 0.99 and 1.06 Å, H3–O_W1_–H4 angle is 100.6º), and W3 site of water (O_W3_ is bonded to H5 and H6 with distances of 1.00 and 1.01 Å, H5–O_W3_–H6 angle is 105.5º). Note that an ideal O–H bond length in H_2_O is approximately 0.97 Å^31^.

The W1 molecule is situated within channel II adjacent to B-tube at the same position (p1, Fig. 4) as W1 in the phase model C1. The shortest H1···O distance is 1.49 Å, whereas H2 is 1.46 Å away from F(B)2. The oxygen site of the molecule has two Ca neighbors, Ca(1A)2 and Ca(2A)1 sites, with the bond distance of 2.31 and 2.76 Å, respectively. Its closest anionic neighbor is F(B)2 which lies within the interatomic distance of 2.44 Å. The W1 molecule is introduced into the [8]-coordinated Ca(1A)2 polyhedron (Supplementary Fig. S4a).The Si(2B)1 tetrahedron, effected by W1, has approximately the same distortion parameters as the same position in the previous model C1 (see Supplementary Table S13).

The W2 molecule is accommodated in channel III of A-tube (position p2, Fig. 4) near Na(A)2 atom (2.56 Å), however, the position of Si(3A)1 is even closer, the W2-Si distance is 1.99 Å. The H3···O bond has a length of 1.76 Å, while the H4···O one is significantly shorter (1.46 Å). The undersaturation of the Si(3A)1 site indicates that the bonds are longer than expected (〈Si–O〉  = 1.68 Å vs. 1.62 Å for natural agrellite), and therefore the Si(3A)1 site appears to have a valence sum that is too low (3.46 vu). However, the oxygen atom of the W2 molecule is so close that it is captured by Si, which becomes [5]-coordinated, to fill its valence saturation (Supplementary Fig. S4b). Therefore, in Supplementary Table S13 it cannot be considered as a usual tetrahedron.

BVS value of 1.53 was obtained for Ca(2B)2, indicating another local structural distortion. [7]-coordinated Ca(2B)2 alternates with the seven-vertex Ca(1A)2 to which it is attached by an edge, and Ca(1B)1 and Ca(2A)2 polyhedra previously connected with Ca(2B)2 by edges are now linked through the vertices. Moreover, in this model the Si(3A)2 tetrahedron is attached to this polyhedron by the edge.

W3 molecule is located in channel III of B-tube (position p3, Fig. 4) closed to the Si(1B)1 tetrahedron (O_W3_–Si = 2.02 Å) and it is coordinated by the Na(B)2 cation (Supplementary Fig. S4a), also located inside this cavity at the intersection of two channels. The H5···O and H6···O distances are 1.65 and 1.58 Å, respectively. The Si(1B)1 tetrahedron displays the lowest BVS (3.54 vu) and high TAV (165.51) and TQE (1.053) (Supplementary Table S13) and reveals a considerable off-side tilt. This strong distortion occurs due to significant elongation of shared Si–O bond lengths (〈Si-O_sh_〉  ~ 1.69 Å) with respect to unshared one (< Si-O_sh_〉  = 1.62 Å). At the same time, BVS of the Na(B)2 position increases due to the close location of the water molecule (Na(B)2-O_W3_ = 2.27 Å vs. 〈Na(B)2-O,O_W3_〉  = 2.54 Å).

The dimensions of the apertures of channel III do not change when water molecules are placed inside it (Table 2). However, this significantly reduces the size of the tetrahedral rings of channel I, in which there is no water molecule. Comparing the current model (C2) with the previous one (C1 with one water molecule in the unit cell), it is obvious that this effect is affected precisely by the addition of 2H_2_O to A- and B-tubes of channel III. The shape of A-tube changes dramatically, and the coordination polyhedra in the environment of the water molecules are strongly distorted.

Provided that W2 and W3 could be located in the indicated positions p2 and p3 inside channel III, they theoretically would be able to move along the extension of the channel due to the reasonable size of its apertures.

The calculated values of the vibrational modes of water molecules in the positions p1-p3 are represented in Table 4 and the simulated IR spectrum is given in Fig. 5d.

#### Agrellite structural model with four H_2_O molecules in the unit cell (NaCa_2_Si_4_O_10_F·1H_2_O): model C3

The calculated positions of O and H atoms of water molecules are shown in Supplementary Fig. S5.

W1 molecule is placed in the interlayer space, between Ca-polyhedral bands and Si-chain of silicate B-tube (channel II). Specifically, the oxygen atom of the molecule coordinates Ca(1A)2 and Ca(2A)1 and adjoins Si(2B)1 and Si(4B)1 (Supplementary Fig. S5a). A similar position (p1, Fig. 4) has already been described in the two previous models (W1 in the models C1 and C2). O_W1_ is bonded to H1 and H2 with the distances of 1.03 and 1.02 Å, respectively (H1-O_W1_-H2 angle is 104.5º). The shortest H1···O distance is 1.50 Å, and H2-F(B)2 bond has a length of 1.46 Å. Examining the Ca(1A)2 and Ca(2A)1 polyhedra reveals slight oversaturation (BVS values of 2.14 and 2.17 vu with 〈cation–anion〉 distance ~ 2.47 Å, Supplementary Table S14).

The W2 molecule is inserted in A-tube of channel III next to Si(3A)1 (2.00 Å) and Na(A)2 (2.51 Å) and close to Si(2A)1 (2.85 Å) and Si(1A)1 (2.97 Å) (Supplementary Fig. S5b). O_W2_-H bond lengths are 0.99 Å (H3) and 1.05 Å (H4; H3–O_W2_–H4 angle is 100.5º). It can be noted a relatively strong hydrogen bond, in accord with the short H···O distance: 1.75 Å (H3) and 1.48 Å (H4); whereas the optimum value typically ranges from 1.85 to 2.00 Å^31^.

Finally, W3 and W4 molecules are localized close to each other (2.49 Å) in B-tube of channel III. The oxygen atom of W3 coordinates Na(B)2 cation, whereas O_W4_ is a vertex of the Na(B)1-polyhedron (O-Na distances are equal to 2.37 and 2.14 Å, respectively) (Supplementary Fig. S5a,c). The O_W3_–H5, O_W3_–H6, O_W4_–H7 and O_W4_–H8 bonds are 1.06, 1.00, 0.97, and 1.03 Å, respectively (H5–O_W3_–H6 angle is 110.1º andH7-O_W4_-H8 angle is 108.8º). Analyzed results of calculation of the H···O distances (2.09, 1.66, 1.93, and 1.53 Å for H5, H6, H7, and H8, respectively) indicate that there is a different environmental impact. The BVS for Na(B)1 rather higher than 1 vu (see Supplementary Table S14). The oversaturation indicates the shortening of Na–O_W4_ distance and, consequently, 〈cation–anion〉 value of the Na-polyhedron (2.54 vs. 2.599 Å for Na(B) polyhedron in natural agrellite, see Supplementary Tables S6 and S14).

W3 has the same position (p3, Fig. 4) as W3 in the structural model C2. It has the same effect on distortion and contributes to a violation of the CN of Si(1B)1 (O_W3_–Si = 1.96 Å). Si(3A)1 also, like Si(1B)1, acquires the configuration of a distorted dipyramid (Supplementary Fig. S5c). Neighbor Si-tetrahedron, Si(2A)1, is also highly distorted with TAV = 113.37, TQE = 1.031, BLD = 1.19% (Supplementary Table S13).

Probably, since water molecules occupy similar positions, the channel aperture sizes correspond to those ones in the structural model C2. The exception is B-tube of channel III, in which two W molecules are localized in the model C3. The channel window dimension is somewhat larger – 7.0 × 3.9 Å vs. 7.0 × 3.5 Å in C2.

The data concerning the simulated IR spectrum are given in Fig. 5e and Table 4.

#### Agrellite structural model with five H_2_O molecules in the unit cell (NaCa_2_Si_4_O_10_F·1.25H_2_O): model C4

The W1 molecule is situated in channel II (B-tube) at the same position p1 as W1 molecules in the models C1, C2 and C3, however, due to the strong distortion of the neighboring channel systems, the external environment of the molecule changes dramatically. The molecule is surrounded by three calcium polyhedra (Ca(1A)2, Ca–O_W1_ = 2.37 Å; Ca(2A)1, Ca–O_W1_ = 2.47 Å; and Ca(1B)2, Ca–O_W1_ = 3.09 Å) and one tetrahedron (Si(2B)1, Si–O_W1_ = 2.89 Å) (Supplementary Fig. S6a). O_W1_–H bond lengths are 1.00 and 1.03 Å for H1 and H2, respectively (H1–O_W1_–H2 angle is 107.2º); H1···O = 1.85 Å and a distance between H2 and F(B)2 is equal to 1.49 Å.

W2 molecule belongs to A-tube of channel III. O_W2_–Na(A)2 bond length is 2.53 Å. The O_W2_–H bond lengths are found to be longer than 0.97 Å: O_W2_–H3 = 0.99 Å and O_W2_–H4 = 1.07 Å; H···O distances are 1.84 and 1.41 Å for H3 and H4, respectively (H3-O_W2_-H4 angle is 101.5º). However, the distance between O_W2_ and Si(3A)1 atoms, as it was noted before in models C2 and C3, is too short (1.93 Å). The distances between Si and O atoms in the tetrahedron are unexpectedly long (1.65–1.79 Å with average 1.74 Å), the lack of the valence saturation (3.04 vu) is compensated by two oxygen atoms of the W2 and W6 molecules, showing a strongly asymmetric out-of-side configuration of coordination polyhedron (Supplementary Fig. S6b). The vertex-shared neighbor Si(4A)1 is also strongly distorted and fivefold coordinated in this model. The average Si–O distances are longer than for other Si-tetrahedra (1.71 Å vs. 1.62–1.65 Å, Supplementary Table S13), as a result, affected by the water incorporation, an additional connection (1.88 Å) with the F(A)1 position is formed (where the oxygen of OH-group may be located).

W4 and W5 are in the B-tube of channel III. However, the W4 molecule has slightly different atomic coordinates and the environment with respect to W4 in the model C3 (position p4, Fig. 4). W4 and W5 molecules are shown in Supplementary Fig. S6a; H7 and H8 bonded to O_W4_ with a distance of 0.97 and 1.01 Å (H7–O_W4_–H8 angle is 106.4º), and O_W5_–H lengths are 0.97 and 1.01 Å for H9 and H10, respectively (H9–O_W5_–H10 angle is 105.8º). The shortest H···O distances are 2.12 and 1.67 Å for H7 and H8, whereas H9···O = 1.89 Å and H10···O = 1.56 Å. The oxygen of W5 is the nearest vertex of the [8]-coordinated Na(B)2 polyhedron (Na–O_W5_ = 2.10 Å, whereas the average Na–O distance for Na(B)2 is equal to 2.48 Å, and BVS = 1.35 vu (Supplementary Table S14). O_W4_ is also one of the eight vertices of this polyhedron (Na–O_W4_ = 2.67 Å). The distance between O_W4_ and O_W5_ is 4.32 Å, and between O_W4_ and the closest Na(B)1 cation is 2.67 Å.

W6 molecule is 2.59 Å away from W2 and hosted in A-tube of channel I. This position p6 is located in the central part of the six-membered tetrahedral ring (Fig. 4, Supplementary Fig. S6b); therefore, the oxygen atom has close distances with Si(3A)1 (2.00 Å), Si(1A)2 (2.38 Å), and longer distances with Si(1A)1 (2.94 Å) and Si(2A)1 (3.05 Å). O_W6_–H11 and O_W6_–H12 lengths are equal to 1.04 and 0.98 Å (H11–O_W6_–H12 angle is 105.4º); H11···O and H12···O distances are 1.46 and 1.96 Å, respectively. The Si(1A)2 tetrahedra volume is larger than the average value for all Si-tetrahedra in the unit cell of the model C4 (2.26 vs. 2.19 Å^3^) and BVS value is correspondingly lower (3.80 vu). In contrast, the other tetrahedron closest to the molecule is critically distorted (Supplementary Fig. S6b). The Si(3A)1 is influenced by the proximity of another water molecule (W2) incorporated into the structure.

Analyzing the dimensions of the channel apertures, it can be concluded that the fifth additional molecule in the structural model does not really change the size of the windows of channels I and III (Table 2). The W6 molecule is fixed in the cavity of channel I. The dimensions of the tetrahedral rings allow it to pass through them, i.e. migrate along the length of the channel. However, the shortest distances between oppositely located oxygen atoms of the polyhedra in B-tube of channel II are greatly reduced (3.4 × 2.7 vs. 4.8 × 2.7 Å in the model C3). This suggests that tube and channel geometry is greatly affected by the water molecule incorporation.

It should be noted, that BVS values for all Ca-polyhedra in the model do not exhibit significant deviations from 2 vu (Supplementary Table S14), the exception is Ca(2B)1, which BVS value is 7% higher than the average valence saturation of Ca-polyhedra.

Figure 5f represents the theoretical IR spectrum, while Table 4 lists the values of vibrational modes for water molecules theoretically located in the model C4.

Obviously, such parameters as bond length distortion and angle variance increase with the increasing of the water content in the unit cell. Supplementary Fig. S7 shows that for Si-tetrahedra strong linear correlation exists between quadratic elongation and angle variance, which gives similar quantitative measures.

#### Agrellite crystal structure stability

The unit cell of agrellite may be considered as composed of chemically distinct units and a lattice-induced strain may be estimated. The complimentary measure of the strain of whole crystal structure is expressed in the global instability index (GII), defined by^32^ as GII = 100%·〈($\sum\limits_{j} {\text{Sij } - \text{ Vi}}$)^2^〉^1/2^, where Sij is experimental bond valence between cation i and anion j and Vi is the cation valence. Values of the GII <5% suggest that little or no strain is presented, and values >20% indicate that structure is so strained as to be unstable^33^.

It is interesting to compare the stability of the initial crystal structure of natural agrellite and simulated H_2_O-containing structural models (Supplementary Table S15). Agrellite itself shows a significantly increased index for Ca and F (21.85 and 20.42%, respectively), but Na, O and Si are still in the medium range. The incorporation of one water molecule (model C1) leads to local structural relaxation, indicating higher stability. Although the GII value for Si slightly increases (10.79%), the indices for Ca and GII_total_ are getting lower (13.98 and 12.14%, respectively). In the initial structure, calcium is the most unstable position; therefore, during the modeling of the structure with one water molecule, it is embedded in the structure as the calcium polyhedron vertex for relaxation of local instability. Insertion of extra ions (W1 molecule) on the interstitial site helps to relax the stretched polyhedral layers, increasing Ca(1A) valence sum.

Three water molecules (model C2) change the GIIs in the direction of increasing. Now the GII value for F slightly exceeds 20%, namely 23.75% (Supplementary Table S15). For the model C3, the value for F also exceeds 20%, and the model C4 has a high value of GII for Na with 20.74% (Supplementary Table S15). In the latter case, significant local relaxation of Ca is noted. Assuming only GII_total_ values, all structures (natural agrellite, models C1-C4) can be considered stable (GII_total_ ranges from 12.14 to 14.72%).

It is noteworthy that the overall stress quantified by the global instability index (GII) significantly varies across the models. The lattice-induced lengthening or shortening of some bonds are compensated by the shortening or lengthening of the unstrained bonds so as to preserve the valence sum rule. The ions may move off-center of its coordination polyhedra, where the bonds formed by these ions are stretched^33^. It is indicated again that the coordination of Ca and Na and the environment of Si-ring is the driving force for the stabilization of the crystal structure model.

## Discussion

The calculated values of the IR spectrum frequencies and types of vibrations for each of the models C1–C4 (Table 4) make it possible to predict the energy value ranges (cm^-1^) of the appearance of peaks attributed to each of the positions of the H_2_O molecule (p1-p6) in the studied structure.

1. *Bending vibrational modes *(B see Table 4).The vibration peaks for the water molecule in position p1 are in the range 1650–1700 cm^−1^, for the molecule W2 the peaks are slightly displaced and are situated approximately between 1580 and 1630 cm^−1^. Bending vibrational mode of the H_2_O molecule in p3 can be found around 1550–1600 cm^−1^. An offset also takes place for the molecule in position p4 (~ 1530–1580 cm^−1^). For positions p5 and p6, the vibrational frequency is approximately 1600–1650 cm^−1^.
2. *Symmetric stretching vibrational modes *(SS, see Table 4). For the H_2_O molecule at position p1, a peak attributed to the SS mode appears on the spectrum in the range 2850–2900 cm^−1^. The models with W2 in the structure have a peak at 3410–3460 cm^−1^. In contrast, W3 is characterized by different vibration frequencies for the models C2 and C3 (~ 3226 and ~ 2747 cm^−1^). The same feature is observed for W4. In the model C3, the peak at about 3222 cm^−1^ corresponds to SS vibrations, and in the model C4 the peak at 3074 cm^−1^ corresponds to this type of vibration. In model C4, the peaks at 2979 and 3559 cm^−1^ are also attributed to the SS vibrational modes of the H_2_O molecules in the positions p5 and p6, respectively.
3. *Asymmetric stretching vibrational modes *(AS, see Table 4). AS vibration modes for the water molecule in the position p1 are attributed to the peak, which can be found in a rather large range of about 2630–2800 cm^−1^. The peak located in the region 2150–2450 cm^−1^ is attributed to AS vibrations of the W2 molecule. For the molecules at the positions p3 and p4, the peak can be seen at 3027and 2300 cm^−1^ in the model C2 and 3758 and 3763 cm^−1^ in the model C3, respectively. In the models C5 and C6, the peak attributed to the AS vibrational modes was observed at 3699 and 2599 cm^−1^, respectively.

Considering the features of the experimental IR spectrum of agrellite the complicated absorption band in the region 2900–3700 cm^−1^ can be separated on peaks attributed to water molecules in different positions using thermal dehydration. Water molecules in different structural voids of agrellite have hydrogen bonds with Si–O–Si tetrahedral tubes. The binding energy of water molecules depends on its position in the host voids. Therefore, the activation energy of water escaping from various positions should also be different. It means that the shape of the band in the region 2900–3700 cm^−1^ could be changed in preheated minerals. The spectra of dehydrated agrellite in the 50–550 °C temperature range are shown in Fig. 6. The intensity of all spectra was normalized on the absorbance of peak at 687 cm^−1^. It is clearly seen, that intensity and shape of peaks corresponding to host Si–O–Si vibrations are not changed. However, the intensity and shape of water attributed peak are varied. The wide peak could be deconvoluted into three Gaussian peaks with maxima at 3065, 3470, 3555 cm^−1^. Dependence of integral absorbance of each peak on heating temperature is given in Supplementary Fig. S8. It is noted, that behavior of the intensity of peaks decreasing is different. It means, that complex water attributed peak in region 2800–3700 cm^−1^ is caused by the absorption of water molecules in different voids positions. The peak at 3470 cm^−1^ corresponds to the water molecule which position is the most stable with respect to heating.

To identify the water positions the ab initio calculation results reported in Table 4 were used. From the calculated values of the vibrational modes of water in various voids of agrellite shown in Table 4, it can be concluded that a wide peak in the region of 3470 cm^−1^ (SS), weak peaks at about 2516 (AS) and 1633 cm^−1^ (B) are associated with the water molecule in the position p2 of channel III (A-tube). A wide peak in the region of 3065 cm^−1^ (AS + SS) and a peak at 1426 cm^−1^ (B) refer to the water molecule in the position p3 of channel III (B-tube).

The nature of the narrow band at about 3555 cm^−1^ belongs to vibrations of the hydroxyl anion in the fluorine position. This is confirmed by calculations given in Fig. 5b. The broadening of this peak is caused by the fact that the agrellite unit cell has two nonequivalent positions of fluorine ions, and according to the calculated data, positions of their absorption bands differ slightly. Their superposition gives the broadened peak observed in the experiment. The hydrogen atom is not involved in H-bonds, and the position of the peak depends on metal–oxygen interaction. The distance between the oxygen and calcium ions can be estimated using the approximation given in^34^. The Ca–O is ~ 2.4 Å, that is close to the distance between fluorine and calcium ions in agrellite (~ 2.47 Å) obtained by single-crystal X-ray diffraction (Supplementary Table S6).

It is noted the rather high temperature of water release when heated, the dehydration mostly takes place over 200–380 °C temperature range (Supplementary Fig. S8). This indicates tightly bounded water molecules within the crystalline space, and not surface water (absorbed).

The analyzed data point out that, most likely, the water molecule is absent in the calculated most energetically favorable position p1 (inside the void of channel II).This fact has an explanation in the features of the crystal structure of the mineral. Thermal water escaping and, apparently, its incorporation depend on the size of the structural channel apertures. According to the results of single-crystal X-ray diffraction, the apertures of channel III are the largest (7.0 × 3.5–3.6 Å), and dimensions of the channel II windows are much smaller (4.6–4.8 × 3.0–3.4 Å). Wherein, the effective channel aperture dimensions are 4.3–0.8 × 3.0–3.4 Å and 1.9–2.1 × 0.3–0.7 Å for channel III and channel II, respectively (Table 2). This suggests that the water molecule cannot pass through the channel II windows due to its too small size, while it could move through the channel III windows.

Despite close values of the relative energies of the crystal structure with a single H_2_O molecule at the positions p2 and p3 (Table 3), the temperatures of the exit of the molecule from the structure differ by more than 150 ºC (Supplementary Fig. S8). The water molecule at the position p3 leaves the structural channel at a higher temperature. Most likely, the difference is due to the different ionic environment of the molecules.

Obviously, water escape occurs during the thermal expansion of the whole structure at the high-temperature range and the extension of the size of the channel apertures sufficient for water molecules. The difficult localization of the small amounts of H_2_O molecules incorporated into phases stable at the temperatures and pressures may indicate the post-crystallization nature of the appearance of water molecules in the structure of Murun agrellite. The lack of water in the agrellite from the Dara-i-Pioz massif^2^, which crystal structure is identical to Murun agrellite, confirms this assumption. The presence of H_2_O molecules in the crystal structure may indicate that aqueous solution participated in the post-crystallization processes of the formation of agrellite from Murun massif.

## Conclusion

The present study was concerned with the crystal structure and water contamination features in agrellite, complex silicate mineral from Murun massif, Russia. Distribution of atoms in the unit cell was determined using single crystal X-ray diffraction and electron microprobe analysis and was confirmed by the results of ab initio calculations.

It was found that agrellite can contain water molecules in its channel system. All possible positions of water molecules in the structural pores were determined. Trivial energetic considerations based on comparing total energies of structures with the ones of a single water molecule in different positions showed that the most favorable site (p1) for the water molecule locates in the channel II of the B-tube between the tetrahedral chain and Ca-polyhedral bands. However, this position was established to be unoccupied in the real crystal structure because of too small apertures of the structural channel. Instead, the experiments on preheated samples indicated that other positions located within channels with larger apertures can be occupied by water molecules: p2 inside the channel III of the A-tube and p3 within the channel III (B-tube). Furthermore, the presence of hydroxyl anions (OH^-^) as a fluorine substitute with an IR-absorption peak at about 3555 cm^−1^ was found.

The applied ab initio method has proven to generate IR spectra of the agrellite being in good agreement with experiments, which provides confidence in the realistic description of location and geometry of water molecules and OH-group that cannot be measured directly by using X-ray techniques.

## Methods

### Chemical analysis

Electron microprobe analysis was carried out on a single crystal of the studied agrellite embedded in epoxy resin, polished and the carbon coated. The same crystal was used for X-ray analysis.

A JEOL JXA-8200 electron microprobe operating at 15 kV accelerating voltage, 5nA sample current, ~ 1 μm spot size, and 40 s counting time was used. Full wavelength-dispersive spectrometry (WDS) mode was employed. The used standards for major, minor, and REE components were: wollastonite (Si), anorthite (Ca, Al), omphacite (Na), F-apatite (F), olivine (Mg), K-feldspar (K), rhodonite (Mn, Zn), fayalite (Fe), celestine (Sr), Zr-jarosite (Zr, Hf), sanbornite (Ba), La-phosphate (La), Ce-phosphate (Ce), Pr-phosphate (Pr), Nd-phosphate (Nd), Sm-phosphate (Sm), Eu-phosphate (Eu), Gd-phosphate (Gd), Dy-phosphate (Dy), Ho-phosphate (Ho), Er-phosphate (Er), Yb-phosphate (Yb), Lu-phosphate (Lu), Sc-phosphate(Sc), ilmenite (Ti), Y-phosphate (Y), Cs-pollucite and pure Cu, V, Cr, Co, Ni, Ge, Nb.

For the conversion from X-ray counts to oxide weight percentages (wt%) a Phi-Rho-Z method was employed as implemented in the Jeol suite of program.

### Structural analysis

The crystal-structure determination was performed with a Bruker AXS X8 APEXII automated diffractometer equipped with a four-circle Kappa goniometer, a CCD detector, and monochromatized Mo*Kα* radiation. Operating conditions were: 50 kV and 30 mA, crystal-to-detector distance of 40 mm. The collection strategy was optimized with the COSMO program in the APEX2 suite package^35^ and the entire Ewald sphere (± *h*, ± *k*, ± *l*) up to θ_max_ ~ 39º was recorded by a combination of several ω and φ rotation sets, with 0.5º scan width and 10–50 s per frame exposure time. The SAINT package was used for the extraction of the reflection intensities and for the correction of the Lorenz-polarization effect^36^. The SADABS software provided for a semi-empirical absorption correction^37^. The XPREP software^38^ was used for calculation of the intensity statistics. The structure refinement was then performed against *F* in the space group *P* $\stackrel{-}{1}$ using the program CRYSTALS^39^. Reflections with *I *> 3σ(*I*) were considered as observed and the refined parameters were: scale factor, atom positions, anisotropic displacement parameters and Ca, Na cations and F anions occupancies. Occupancies for Si and O atoms were constrained to 1. Ionized X-ray scattering curves were used for non-tetrahedral cations and anions, whereas ionized *vs* neutral curves were employed for Si and O atoms^40^. Initial fractional coordinates were taken from^2^. Atom labeling is after^4^ and^2^. The final fully anisotropic refinement converged to *R* = 3.94% (*Rw* = 4.04%). The CIF is deposited with the Cambridge Crystallographic Data Centre (CCDC 2011870) and is also available from the authors.

### Infrared spectroscopy

The Infrared (IR) absorption spectrum of agrellite powder in a KBr pellet was recorded by a Fourier-transform spectrometer Simex FT-801 in the spectral range from 520 to 5300 cm^−1^ with spectra resolution 0.5 cm^−1^. The spectrum was taken from a tablet sample shaped as about 0.4 mm thick tablet of 4 mm in diameter.

The complicated absorption band attributed to water molecules in different positions could be separated using a thermal dehydration procedure. To avoid the effect of water adsorbed by KBr all absorption spectra were measured relative to the pure KBr that was preheated at the same temperature. The measurement procedure was performed as follows: (1) the mixture of agrellite and preliminary dried KBr powder was pressed into a transparent tablet; (2) the IR-absorption spectrum was measured with respect to the dried KBr transparent tablet; (3) then, the mixture was heated to a certain temperature and maintained at this temperature for 3 min; (4) similar heating and maintaining operations were performed for pure dried KBr powder; (5) then the mixture and pure KBr powder were cooled down to room temperature; (6) the IR-absorption spectrawere measured again. These steps were repeated during the heating from 130 ºC up to 530 ºC.

### Calculation details

The calculations were performed using VASP ab initio code^41^, with pseudopotential approach and plane wave basis sets. Exchange and correlation were expressed in terms of PBEsol functional^42^. Energy cutoff for plane wave basis sets was 400 eV. The Brillioun zone sampling was performed using gamma-centered 2 × 1 × 2 Monkhorst–Pack meshes. The following electrons were treated as valent: 4*s*^2^ for Ca, 3*s*^1^ for Na, 3*s*^2^, 3*p*^2^ for Si, 2*s*^2^, 2*p*^5^ for F and 2*s*^2^, 2*p*^4^ for O, 1*s*^1^ for H. Volume and unit cell parameters of agrellite were fixed at their experimental values.

To incorporate water molecules into the agrellite crystal structure its unit cell was scanned in three dimensions until an empty sphere of radii *R* was found. A water molecule was placed at the center of the sphere. The water content in the simulation unit cells C1, C2, C3, C4 is 1.1, 3.3, 4.4, and 5.3 wt%, respectively. The choice of simulation cell needs to be addressed. Any simulated water-containing structure contains an H_2_O excess with respect to experimental concentration. This is a result of a compromise between computational time and accuracy. Due to periodic boundary conditions applied to unit cell the maximal spacing between H_2_O molecules is equal to the size of the smallest lattice vector (*c* = 6.983 Å). We estimated the error introduced into IR-spectra by excessive structural relaxation as well as H_2_O–H_2_O interaction by comparing calculations conducted for single water molecule (p1) within the unit cell and for the same molecule within the cell doubled along the vector *c*. This corresponds to 0.57 wt% modeled concentration. We found that the greatest shift of an absorption band attributed to H_2_O is 5.8%, while the average shift between three bands is about 3%. A similar calculation conducted for water molecule (p1) within the cell doubled along the vector *a* (7.763 Å) showed that the greatest shift is 2.9% while the average shift is only 1.3%. Thus, we expect that the greatest error introduced to the peak positions by using small-volume simulation cells would not exceed 6%, while would be lower in many cases.

Phonon calculations require very optimized geometry, therefore positions of all atoms in the cells were relaxed until maximal force acting on atom became less than 0.001 eV/Å. Subsequent calculations of phonon frequencies and eigenvectors were straightforward and guided by procedures implemented in the Phonopy auxiliary code^43^. The Born effective charges were calculated within density functional perturbation theory^44^.

The infrared spectra were simulated with Phonopy-Spectroscopy tool^45^.

### Distortion parameters calculation

1. Bond length distortion is calculated as BLD = 100/*n*· ${\sum }_{i=1}^{n} \left| (\mathrm{M} - \mathrm{O})i - \langle \mathrm{M} - \mathrm{O} \rangle i \right | / \langle \mathrm{M} - \mathrm{O} \rangle$(%), where *n* is the number of bonds and (M–O) is the central cation-oxygen length^46^;
2. Edge length distortion is calculated as ELD = 100/*n* ${\sum }_{i=1}^{n}|(\mathrm{O} - \mathrm{O})i - \langle \mathrm{O} - \mathrm{O} \rangle i | / \langle \mathrm{O}- \mathrm{O} \rangle$(%), where *n* is the number of bonds and (O–O) is the oxygen–oxygen edge (and also, in our case, oxygen-fluorine and fluorine-fluorine) length^46^;
3. Tetrahedral angle variance is calculated as TAV = ${\sum }_{\mathsf{i}=1}^{6}{\left({\theta }_{\mathsf{i}} - 109.47\right)}^{2}/5$, where *θ* is individual bond angle^47^;
4. Tetrahedral quadratic elongation is calculated as TQE = ${\sum }_{i=1}^{4}({\mathrm{I}i/\mathrm{I}o)}^{2}/4$, where I*o* is the center to vertex distance for an undistorted tetrahedron whose volume is equal to that of the distorted tetrahedron with bond length I*i*^47^;
5. Octahedral angle variance is calculated as OAV = ${\sum }_{i=1}^{12}({{\theta }_{\mathsf{i}}-90)}^{2}/11$, where *θi *is individual bond angle^47^;
6. Octahedral quadratic elongation is calculated as OQE = ${\sum }_{i=1}^{6}({\mathrm{I}i/\mathrm{I}o)}^{2}/6$, where I*o* is the center to vertex distance for an undistorted octahedron whose volume is equal to that of the distorted octahedron with bond length I*i*^47^.

Bond length and edge length distortion (BLD and ELD) are measure of dispersion of the individual bond lengths and edge length, i.e., a large value indicates dispersed bonds, while low ones indicate that the bonds are closely grouped around an average value. The bond angle variance (TAV and OAV) is equal to 0 for a regular polyhedron and is > 0 for a distorted polyhedron. The quadratic elongation (TQE and OQE) is dimensionless and equal to 1 for a regular polyhedron while it is > 1 for a distorted polyhedron.

## Supplementary information

Supplementary Information.

## Data Availability

All data supporting the findings of this study are available in Supplementary Material.
