# Supplementary material for: Structural and vibrational properties of agrellite
Source: Sci Rep. 2020 Sep 23;10:15569. doi: 10.1038/s41598-020-72631-1 (PMC7511943; doi:10.1038/s41598-020-72631-1)
Supplement: Supplementary file 1 — Supplementary Information. [file 41598_2020_72631_MOESM1_ESM.doc]

Supplementary Materials

**Structural and vibrational properties of agrellite**

**Ekaterina Kaneva*, Alexandr Bogdanov* & Roman Shendrik***

Vinogradov Institute of Geochemistry, Siberian Branch of the Russian Academy of Sciences, Irkutsk, 664033, Russia.

*e-mail: [kev604@mail.ru](mailto:kev604@mail.ru)

*e-mail: [alex.bogdanov2012@gmail.com](mailto:alex.bogdanov2012@gmail.com)

*e-mail: roshen@yandex.ru

**TABLES**

**Table S1**. Average chemical composition (wt%) and atomic proportions (apfu), calculated on the basis of Si = 4, of the studied agrellite crystal

|  | Wt.% | Range | Atoms per formula unit | |
| --- | --- | --- | --- | --- |
| SiO_2_ | 59.9(9) | 58.23 - 60.12 | Si | 4.000 |
| Na_2_O | 7.57(9) | 7.18 - 8.04 | Na | 0.980 |
| MgO | 0.05(1) | 0.033 - 0.053 | Mg | 0.005 |
| K_2_O | 0.29(9) | 0.203 - 0.417 | K | 0.025 |
| CaO | 26.6(3) | 25.5 - 27.1 | Ca | 1.903 |
| MnO | 0.09(2) | 0.07 - 0.12 | Mn | 0.006 |
| FeO | 0.10(1) | 0.09 - 0.12 | Fe | 0.006 |
| CuO | 0.02(1) | 0.01 - 0.04 | Cu | 0.001 |
| SrO | 1.06(6) | 0.97 - 1.08 | Sr | 0.042 |
| ZrO_2_ | 0.05(2) | 0.04 - 0.08 | Zr | 0.002 |
| BaO | 0.06(5) | 0.01 - 0.11 | Ba | 0.002 |
| La_2_O_3_ | 0.15(6) | 0.07 - 0.17 | La | 0.004 |
| Ce_2_O_3_ | 0.06(4) | 0.03 - 0.12 | Ce | 0.002 |
| Eu_2_O_3_ | 0.16(4) | 0.11 - 0.18 | Eu | 0.004 |
| Dy_2_O_3_ | 0.16(4) | 0.10 - 0.26 | Dy | 0.004 |
| Er_2_O_3_ | 0.14(2) | 0.07 - 0.17 | Er | 0.003 |
| Yb_2_O_3_ | 0.05(4) | 0.04 - 0.11 | Yb | 0.001 |
| F | 3.6(3) | 3.5 - 4.0 | F | 0.760 |
|  | 100.11 |  |  |  |
| O=F | 1.516 |  |  |  |
| Sum | 98.594 |  |  |  |
|  |  |  |  |  |
| H_2_O corresponds to OH group ^(1)^ | 1.015 |  | OH ^(1)^ | 0.240 |
| H_2_O corresponds to molecular water ^(2)^ | 0.391 |  | H_2_O ^(2)^ | 0.087 |

1. Calculated using a single-crystal X-ray diffraction data refinement
2. Calculated based on “Total” = 100%

Al_2_O_3_, Sc_2_O_3_, TiO_2_, V_2_O_3_, Cr_2_O_3_, CoO, NiO, ZnO, GeO_2_, Y_2_O_3_, Nb_2_O_5_, Cs_2_O, Pr_2_O_3_, Nd_2_O_3_, Sm_2_O_3_, Gd_2_O_3_, Ho_2_O_3_, Lu_2_O_3_, HfO_2_ – below detection limit

**Table S2.** Selected data on single crystals, data collection and structure refinement parameters of the studied agrellite

| **Crystal data** | |
| --- | --- |
| *a* (Å)  *b* (Å)  *c* (Å) | 7.7627(4)  18.959(1)  6.9829(4) |
| α (°)  β (°)  γ (°) | 89.775(3)  116.573(2)  94.342(3) |
| *V* (Å^3^) | 915.97(5) |
| *Z* | 4 |
| Crystal dimensions (mm) | 0.049 × 0.058 × 0.742 |
| **Data collection** | |
| Independent reflections | 9222 |
| R_merging_ [R_(int)_] (%) | 3.70 |
| h_min_, h_max_ | -13, 10 |
| k_min_, k_max_ | -30, 33 |
| l_min_, l_max_ | -7, 11 |
| **Refinement** |  |
| Space group | *P*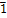 |
| Reflections used in the refinement (I > 3σ_(I)_) | 4634 |
| N. of refined parameters | 361 |
| *R*^a^ [on *F*] (%) | 3.94 |
| *R*_w_^b^ [on *F*] (%) | 4.04 |
| Goof^c^ | 1.135 |
| Δρ_min_/Δρ_max_ (e^-^/Å^3^) | -0.72/0.71 |

**^a^***R* = Σ[|F_o_| – |F_c_|]/Σ|F_o_|.

^b^ *R***_w_** = [Σ[*w*(*F_o_*^2^-*F_c_*^2^)^2^]/Σ[*w*(*F*_o_^2^)^2^]]^1/2^; *w* = Chebyshev optimized weights.

^c^ Goodness-of-fit = [Σ[*w*(*F_o_*^2^-*F_c_*^2^)^2^]/(*N-p*]^1/2^, where N and p are the number of reflections and parameters, respectively.

**Table S3.** Crystallographic coordinates, occupancies and equivalent/isotropic atomic displacement parameters (Å^2^) of agrellite.

| Site | Atom type | x/a | y/b | z/c | Occ. | Ueq |
| --- | --- | --- | --- | --- | --- | --- |
| Ca(1A) | Ca^2+^ | 0.0029(1) | 0.21506(5) | 0.9995(1) | 1.025(1) | 0.0122 |
| Ca(1B) | Ca^2+^ | 0.5441(1) | 0.28438(4) | 0.0211(1) | 1.021(1) | 0.0096 |
| Ca(2A) | Ca^2+^ | 0.4525(1) | 0.72051(4) | 0.4772(1) | 1.005(1) | 0.0092 |
| Ca(2B) | Ca^2+^ | -0.0022(1) | 0.78162(4) | 0.4968(1) | 1.091(1) | 0.0116 |
| Na(A) | Na^+^ | 0.2355(2) | 0.9908(1) | 0.8645(3) | 1.021(1) | 0.0248 |
| Na(B) | Na^+^ | 0.2604(2) | 0.5023(1) | 0.1350(4) | 1.001(1) | 0.0262 |
| Si(1A) | Si^4+^, Si | 0.2094(1) | 0.93099(5) | 0.3538(2) | 1.0000(7) | 0.0078 |
| Si(1B) | Si^4+^, Si | 0.3092(1) | 0.56837(5) | 0.6556(2) | 1.0000(7) | 0.0080 |
| Si(2A) | Si^4+^, Si | 0.4861(2) | 0.87798(6) | 0.2118(2) | 1.0000(7) | 0.0084 |
| Si(2B) | Si^4+^, Si | 0.0250(1) | 0.61934(6) | 0.7933(2) | 1.0000(7) | 0.0079 |
| Si(3A) | Si^4+^, Si | 0.1666(1) | 0.09005(5) | 0.3353(2) | 1.0000(7) | 0.0081 |
| Si(3B) | Si^4+^, Si | 0.6742(1) | 0.58999(5) | 0.3411(2) | 1.0000(7) | 0.0075 |
| Si(4A) | Si^4+^, Si | 0.4872(1) | 0.87742(6) | 0.7721(2) | 1.0000(7) | 0.0080 |
| Si(4B) | Si^4+^, Si | 0.0218(2) | 0.61967(6) | 0.2342(2) | 1.0000(7) | 0.0080 |
| F(A) | F^-^ | 0.7607(3) | 0.7607(1) | 0.1276(4) | 0.997(1) | 0.0183 |
| F(B) | F^-^ | 0.2355(3) | 0.2450(1) | 0.3652(4) | 1.002(1) | 0.0184 |
| O(1A) | O^2-^, O | 0.3525(4) | 0.9353(1) | 0.6128(4) | 1.0000(7) | 0.0134 |
| O(1B) | O^2-^, O | 0.1654(4) | 0.5645(1) | 0.3979(4) | 1.0000(7) | 0.0127 |
| O(2A) | O^2-^, O | 0.1024(3) | 0.0047(1) | 0.3019(5) | 1.0000(7) | 0.0119 |
| O(2B) | O^2-^, O | 0.5876(3) | 0.5061(1) | 0.2957(4) | 1.0000(7) | 0.0093 |
| O(3A) | O^2-^, O | 0.3475(4) | 0.9355(1) | 0.2317(5) | 1.0000(7) | 0.0126 |
| O(3B) | O^2-^, O | 0.1737(4) | 0.5644(1) | 0.7817(5) | 1.0000(7) | 0.0135 |
| O(4A) | O^2-^, O | 0.0642(4) | 0.8633(2) | 0.2832(5) | 1.0000(7) | 0.0130 |
| O(4B) | O^2-^, O | 0.4560(4) | 0.6360(2) | 0.7251(5) | 1.0000(7) | 0.0103 |
| O(5A) | O^2-^, O | 0.3956(5) | 0.7990(2) | 0.1906(6) | 1.0000(7) | 0.0109 |
| O(5B) | O^2-^, O | 0.1090(4) | 0.6988(2) | 0.8139(6) | 1.0000(7) | 0.0110 |
| O(6A) | O^2-^, O | 0.5128(4) | 0.9063(1) | 0.0045(4) | 1.0000(7) | 0.0106 |
| O(6B) | O^2-^, O | 0.9970(4) | 0.5912(1) | 0.0015(4) | 1.0000(7) | 0.0130 |
| O(7A) | O^2-^, O | 0.7017(4) | 0.8929(2) | 0.4097(5) | 1.0000(7) | 0.0156 |
| O(7B) | O^2-^, O | 0.8134(4) | 0.5995(2) | 0.5958(4) | 1.0000(7) | 0.0149 |
| O(8A) | O^2-^, O | 0.9739(4) | 0.1292(2) | 0.2399(5) | 1.0000(7) | 0.0105 |
| O(8B) | O^2-^, O | 0.5005(4) | 0.6385(2) | 0.2577(5) | 1.0000(7) | 0.0099 |
| O(9A) | O^2-^, O | 0.7036(4) | 0.8909(2) | 0.7904(5) | 1.0000(7) | 0.0157 |
| O(9B) | O^2-^, O | 0.8076(4) | 0.6025(2) | 0.2176(5) | 1.0000(7) | 0.0152 |
| O(10A) | O^2-^, O | 0.3931(4) | 0.7984(2) | 0.7040(6) | 1.0000(7) | 0.0104 |
| O(10B) | O^2-^, O | 0.1067(4) | 0.6992(2) | 0.2953(6) | 1.0000(7) | 0.0105 |

**Table S4.** Anisotropic atomic displacement parameters (Å^2^) of agrellite.

| Site | Atom type | U11 | U22 | U33 | U23 | U13 | U12 |
| --- | --- | --- | --- | --- | --- | --- | --- |
| Ca(1A) | Ca^2+^ | 0.0169(3) | 0.0108(3) | 0.0104(3) | -0.0007(2) | 0.0075(3) | 0.0012(2) |
| Ca(1B) | Ca^2+^ | 0.0112(3) | 0.0089(3) | 0.0093(3) | -0.0010(2) | 0.0053(2) | 0.0003(2) |
| Ca(2A) | Ca^2+^ | 0.0100(3) | 0.0098(3) | 0.0085(3) | -0.0005(2) | 0.0048(2) | 0.0007(2) |
| Ca(2B) | Ca^2+^ | 0.0156(3) | 0.0102(3) | 0.0115(3) | -0.0004(2) | 0.0082(2) | 0.0012(2) |
| Na(A) | Na^+^ | 0.0147(7) | 0.029(1) | 0.029(1) | -0.0011(8) | 0.0069(6) | 0.0001(6) |
| Na(B) | Na^+^ | 0.0128(7) | 0.032(1) | 0.031(1) | 0.0000(8) | 0.0065(7) | 0.0001(6) |
| Si(1A) | Si^4+^, Si | 0.0094(3) | 0.0064(4) | 0.0083(4) | -0.0008(3) | 0.0044(3) | 0.0005(3) |
| Si(1B) | Si^4+^, Si | 0.0083(3) | 0.0070(4) | 0.0095(4) | -0.0004(3) | 0.0045(3) | 0.0010(3) |
| Si(2A) | Si^4+^, Si | 0.0110(4) | 0.0074(4) | 0.0074(5) | -0.0012(3) | 0.0049(3) | -00003(3) |
| Si(2B) | Si^4+^, Si | 0.0088(4) | 0.0092(4) | 0.0066(5) | -0.0008(3) | 0.0043(3) | 0.0006(3) |
| Si(3A) | Si^4+^, Si | 0.0083(3) | 0.0086(4) | 0.0076(4) | -0.0005(3) | 0.0037(3) | 0.0008(3) |
| Si(3B) | Si^4+^, Si | 0.0084(3) | 0.0056(4) | 0.0090(4) | 0.0000(3) | 0.0041(3) | 0.0010(3) |
| Si(4A) | Si^4+^, Si | 0.0102(4) | 0.0086(4) | 0.0067(5) | -0.0004(3) | 0.0050(3) | 0.0004(3) |
| Si(4B) | Si^4+^, Si | 0.0085(3) | 0.0081(4) | 0.0081(5) | -0.0007(3) | 0.0045(3) | 0.0004(3) |
| F(A) | F^-^ | 0.0123(9) | 0.018(1) | 0.021(1) | 0.0004(9) | 0.0050(8) | 0.0000(8) |
| F(B) | F^-^ | 0.0108(9) | 0.017(1) | 0.024(1) | -0.0027(9) | 0.0053(8) | -0.0001(8) |
| O(1A) | O^2-^, O | 0.016(1) | 0.011(1) | 0.010(1) | -0.0001(9) | 0.024(8) | 0.0038(8) |
| O(1B) | O^2-^, O | 0.017(1) | 0.010(1) | 0.009(1) | 0.0015(9) | 0.0035(8) | 0.0029(8) |
| O(2A) | O^2-^, O | 0.0102(9) | 0.007(1) | 0.018(1) | -0.0015(9) | 0.0052(8) | 0.0017(7) |
| O(2B) | O^2-^, O | 0.011(1) | 0.006(1) | 0.012(1) | 0.0004(8) | 0.0053(8) | 0.0015(7) |
| O(3A) | O^2-^, O | 0.017(1) | 0.009(1) | 0.017(1) | -0.0008(9) | 0.0121(9) | 0.0003(8) |
| O(3B) | O^2-^, O | 0.017(1) | 0.011(1) | 0.019(1) | 0.0005(9) | 0.013(1) | 0.0045(8) |
| O(4A) | O^2-^, O | 0.013(1) | 0.012(1) | 0.015(1) | -0.002(1) | 0.008(1) | -0.0024(9) |
| O(4B) | O^2-^, O | 0.012(1) | 0.007(1) | 0.011(1) | 0.0001(8) | 0.0049(9) | -0.0003(8) |
| O(5A) | O^2-^, O | 0.017(1) | 0.009(1) | 0.009(1) | 0.001(1) | 0.008(1) | 0.000(1) |
| O(5B) | O^2-^, O | 0.009(1) | 0.008(1) | 0.014(2) | -0.002(1) | 0.005(1) | -0.0008(9) |
| O(6A) | O^2-^, O | 0.016(1) | 0.010(1) | 0.007(1) | -0.0004(8) | 0.0056(8) | 0.0018(8) |
| O(6B) | O^2-^, O | 0.018(1) | 0.012(1) | 0.012(1) | -0.0030(9) | 0.0100(9) | -0.0029(8) |
| O(7A) | O^2-^, O | 0.014(1) | 0.022(1) | 0.010(1) | -0.003(1) | 0.0049(8) | 0.0011(9) |
| O(7B) | O^2-^, O | 0.015(1) | 0.015(1) | 0.009(1) | -0.0039(9) | 0.0004(8) | 0.0002(8) |
| O(8A) | O^2-^, O | 0.009(1) | 0.009(1) | 0.010(1) | -0.0014(8) | 0.0018(8) | 0.0009(8) |
| O(8B) | O^2-^, O | 0.012(1) | 0.009(1) | 0.010(1) | 0.0002(8) | 0.0045(9) | 0.0047(8) |
| O(9A) | O^2-^, O | 0.016(1) | 0.018(1) | 0.017(1) | 0.000(1) | 0.0113(9) | -0.0026(9) |
| O(9B) | O^2-^, O | 0.013(1) | 0.020(1) | 0.017(1) | 0.002(1) | 0.0106(9) | 0.0005(9) |
| O(10A) | O^2-^, O | 0.011(1) | 0.010(2) | 0.011(1) | -0.002(1) | 0.006(1) | 0.000(1) |
| O(10B) | O^2-^, O | 0.013(1) | 0.006(1) | 0.013(2) | -0.001(1) | 0.006() | 0.0000(9) |

**Table S5.** Selected bond distances (Å), angles (º), volumes (Å^3^) and distortion parameters for silicate tubes tetrahedra of the studied agrellite.

| **Si(1A)- and Si(1B)-tetrahedra** | | | | | | | | |
| --- | --- | --- | --- | --- | --- | --- | --- | --- |
|  | A | B |  | A | B |  | A | B |
| Si1-O1 | 1.644(3) | 1.637(3) | O1-Si1-O2 | 105.4(2) | 105.0(1) | TAV | 13.627 | 15.043 |
| Si1-O2 | 1.636(3) | 1.635(3) | O1-Si1-O3 | 107.3(2) | 107.8(2) | TQE | 1.003 | 1.003 |
| Si1-O3 | 1.639(4) | 1.644(4) | O1-Si1-O4 | 112.5(2) | 112.3(2) | BLD (%) | 1.751 | 1.565 |
| Si1-O4 | 1.564(3) | 1.571(3) | O2-Si1-O3 | 105.5(2) | 105.2(1) | ELD (%) | 0.883 | 1.134 |
| < Si1-O> | 1.621(7) | 1.622(7) | O2-Si1-O4 | 113.2(2) | 113.8(1) |  |  |  |
|  |  |  | O3-Si1-O4 | 112.3(2) | 112.2(2) | Volume | 2.177 | 2.179 |
| **Si(2A)- and Si(2B)-tetrahedra** | | | | | | | | |
|  | A | B |  | A | B |  | A | B |
| Si2-O3 | 1.636(4) | 1.641(4) | O3-Si2-O5 | 112.5(2) | 111.8(2) | TAV | 34.319 | 37.661 |
| Si2-O5 | 1.583(3) | 1.577(4) | O3-Si2-O6 | 101.4(1) | 101.7(2) | TQE | 1.008 | 1.008 |
| Si2-O6 | 1.635(3) | 1.644(3) | O3-Si2-O7 | 108.6(2) | 108.2(2) | BLD (%) | 1.165 | 1.358 |
| Si2-O7 | 1.629(3) | 1.620(2) | O5-Si2-O6 | 115.5(2) | 114.8(2) | ELD (%) | 2.370 | 2.270 |
| < Si2-O> | 1.621(7) | 1.621(7) | O5-Si2-O7 | 114.3(2) | 116.3(2) |  |  |  |
|  |  |  | O6-Si2-O7 | 103.4(2) | 102.7(2) | Volume | 2.159 | 2.158 |
| **Si(3A)- and Si(3B)-tetrahedra** | | | | | | | | |
|  | A | B |  | A | B |  | A | B |
| Si3-O2 | 1.642(3) | 1.654(3) | O2-Si3-O7 | 107.8(2) | 106.5(2) | TAV | 5.536 | 9.173 |
| Si3-O7 | 1.627(3) | 1.618(3) | O2-Si3-O8 | 106.9(2) | 109.1(1) | TQE | 1.001 | 1.002 |
| Si3-O8 | 1.583(3) | 1.576(3) | O2-Si3-O9 | 108.1(2) | 106.7(2) | BLD (%) | 1.142 | 1.290 |
| Si3-O9 | 1.628(4) | 1.623(4) | O7-Si3-O8 | 112.7(2) | 113.7(2) | ELD (%) | 0.689 | 0.674 |
| < Si3-O> | 1.620(7) | 1.618(7) | O7-Si3-O9 | 109.2(2) | 108.0(2) |  |  |  |
|  |  |  | O8-Si3-O9 | 111.9(2) | 112.5(2) | Volume | 2.178 | 2.166 |
| **Si(4A)- and Si(4B)-tetrahedra** | | | | | | | | |
|  | A | B |  | A | B |  | A | B |
| Si4-O1 | 1.630(3) | 1.634(3) | O1-Si4-O6 | 101.3(1) | 102.2(2) | TAV | 34.321 | 31.480 |
| Si4-O6 | 1.635(3) | 1.637(3) | O1-Si4-O9 | 108.5(2) | 108.5(2) | TQE | 1.008 | 1.007 |
| Si4-O9 | 1.627(4) | 1.622(4) | O1-Si4-O10 | 112.4(2) | 112.2(2) | BLD (%) | 0.895 | 1.205 |
| Si4-O10 | 1.592(3) | 1.579(3) | O6-Si4-O9 | 103.5(2) | 103.2(2) | ELD (%) | 2.440 | 2.184 |
| < Si4-O> | 1.621(7) | 1.618(7) | O6-Si4-O10 | 115.5(2) | 114.7(2) |  |  |  |
|  |  |  | O9-Si4-O10 | 114.3(2) | 114.9(2) | Volume | 2.160 | 2.151 |

**Table S6.** Selected bond distances (Å), angles (º), volumes (Å^3^) and distortion parameters for polyhedra of the studied agrellite.

| **Ca(1A) polyhedron** | | **Ca(2B) polyhedron** | |  | **Ca(1A)** | **Ca(2B)** |
| --- | --- | --- | --- | --- | --- | --- |
| Ca(1A)-O(4A) | 2.316(4) | Ca(2B)-O(4A) | 2.332(4) | <Ca-O> | 2.584(9) | 2.615(9) |
| Ca(1A)-O(5A) | 2.758(4) | Ca(2B)-O(10A) | 2.738(3) | <Ca-F> | 2.401(4) | 2.422(4) |
| Ca(1A)-O(5B) | 2.522(4) | Ca(2B)-O(5B) | 2.565(4) | <Ca-O,F> | 2.538(9) | 2.567(9) |
| Ca(1A)-O(8A) | 2.404(4) | Ca(2B)-O(8A) | 2.428(4) |  |  |  |
| Ca(1A)-O(9A) | 3.010(3) | Ca(2B)-O(7A) | 3.094(3) | BLD (%) | 6.811 | 6.806 |
| Ca(1A)-O(10B) | 2.494(4) | Ca(2B)-O(10B) | 2.533(4) | ELD (%) | 9.611 | 9.882 |
| Ca(1A)-F(A) | 2.386(3) | Ca(2B)-F(A) | 2.417(2) | Volume | 25.825 | 26.810 |
| Ca(1A)-F(B) | 2.416(2) | Ca(2B)-F(B) | 2.426(3) |  |  |  |
| **Ca(2A) octahedron** | | **Ca(1B) octahedron** | |  | **Ca(2A)** | **Ca(1B)** |
| Ca(2A)-O(4B) | 2.346(3) | Ca(1B)-O(4B) | 2.333(4) | <Ca-O> | 2.374(7) | 2.367(7) |
| Ca(2A)-O(5A) | 2.388(4) | Ca(1B)-O(5A) | 2.378(4) | <Ca-O,F> | 2.347(8) | 2.342(8) |
| Ca(2A)-O(8B) | 2.350(4) | Ca(1B)-O(8B) | 2.345(3) |  |  |  |
| Ca(2A)-O(10A) | 2.382(4) | Ca(1B)-O(10A) | 2.375(4) | OAV | 28.501 | 43.214 |
| Ca(2A)-O(10B) | 2.402(3) | Ca(1B)-O(5B) | 2.404(3) | OQE | 1.008 | 1.012 |
| Ca(2A)-F(B) | 2.211(2) | Ca(1B)-F(A) | 2.216(2) | BLD (%) | 1.932 | 1.917 |
|  |  |  |  | ELD (%) | 3.295 | 4.250 |
|  |  |  |  | Volume | 17.033 | 16.840 |
| **Na(A) polyhedron** | | **Na(B) polyhedron** | |  | **Na(A)** | **Na(B)** |
| Na(A)-O(1A) | 2.571(4) | Na(B)-O(1B) | 2.582(4) | <Na-O> | 2.677(9) | 2.599(9) |
| Na(A)-O(2A) | 2.355(3) | Na(B)-O(2B) | 2.267(3) |  |  |  |
| Na(A)-O(3A) | 2.560(4) | Na(B)-O(3B) | 2.556(4) | BLD (%) | 8.062 | 7.297 |
| Na(A)-O(6A) | 2.510(3) | Na(B)-O(6B) | 2.413(3) | ELD (%) | 14.518 | 14.420 |
| Na(A)-O(6A’) | 2.605(3) | Na(B)-O(6B’) | 2.588(3) | Volume | 27.755 | 27.351 |
| Na(A)-O(7A) | 3.067(4) | Na(B)-O(7B) | 2.891(4) |  |  |  |
| Na(A)-O(8A) | 2.597(3) | Na(B)-O(8B) | 2.955(3) |  |  |  |
| Na(A)-O(9A) | 3.150(4) | Na(B)-O(9B) | 3.001(4) |  |  |  |

**Table 7.** Bond-valence sum (BVS) and coordination numbers (CN) of the cation and anion structural positions for the agrellite.

| Site | BVS | Site | BVS | CN | Site | BVS | CN |
| --- | --- | --- | --- | --- | --- | --- | --- |
| Ca(1A) | 1.759 | O(1A) | 2.059 | 3 | O(6A) | 2.203 | 4 |
| Ca(1B) | 1.991 | O(1B) | 2.063 | 3 | O(6B) | 2.216 | 4 |
| Ca(2A) | 1.969 | O(2A) | 2.133 | 3 | O(7A) | 2.072 | 4 |
| Ca(2B) | 1.637 | O(2B) | 2.154 | 3 | O(7B) | 2.127 | 3 |
| Na(A) | 0.905 | O(3A) | 2.060 | 3 | O(8A) | 1.805 | 4 |
| Na(B) | 0.959 | O(3B) | 2.036 | 3 | O(8B) | 1.863 | 4 |
| Si(1A) | 4.049 | O(4A) | 1.889 | 3 | O(9A) | 2.080 | 4 |
| Si(1B) | 4.035 | O(4B) | 1.841 | 3 | O(9B) | 2.053 | 3 |
| Si(2A) | 4.040 | O(5A) | 1.860 | 4 | O(10A) | 1.848 | 4 |
| Si(2B) | 4.088 | O(5B) | 1.831 | 4 | O(10B) | 1.875 | 4 |
| Si(3A) | 4.048 |  |  |  | F(A) | 0.805 | 3 |
| Si(3B) | 4.076 |  |  |  | F(B) | 0.787 | 3 |
| Si(4A) | 4.035 |  |  |  |  |  |  |
| Si(4B) | 4.069 |  |  |  |  |  |  |

CN for Ca and Na polyhedra are reported in Table S6, CN for Si = 4.

**Table S8.** Calculated and experimental vibrational modes of SiO_4_ in agrellite. SS - symmetric stretching, AS – asymmetric stretching, B – bending. Due to low symmetry, assignment is approximate (vibrations with high changes in Si-O distances are related to as SA/SS, while vibrations with slight changes in Si-O distances and high changes in angles are assigned to B-type). Sign “-- “ states for (SiO_4_)s which don’t contribute to a particular mode.

| **Agrellite unit cell**  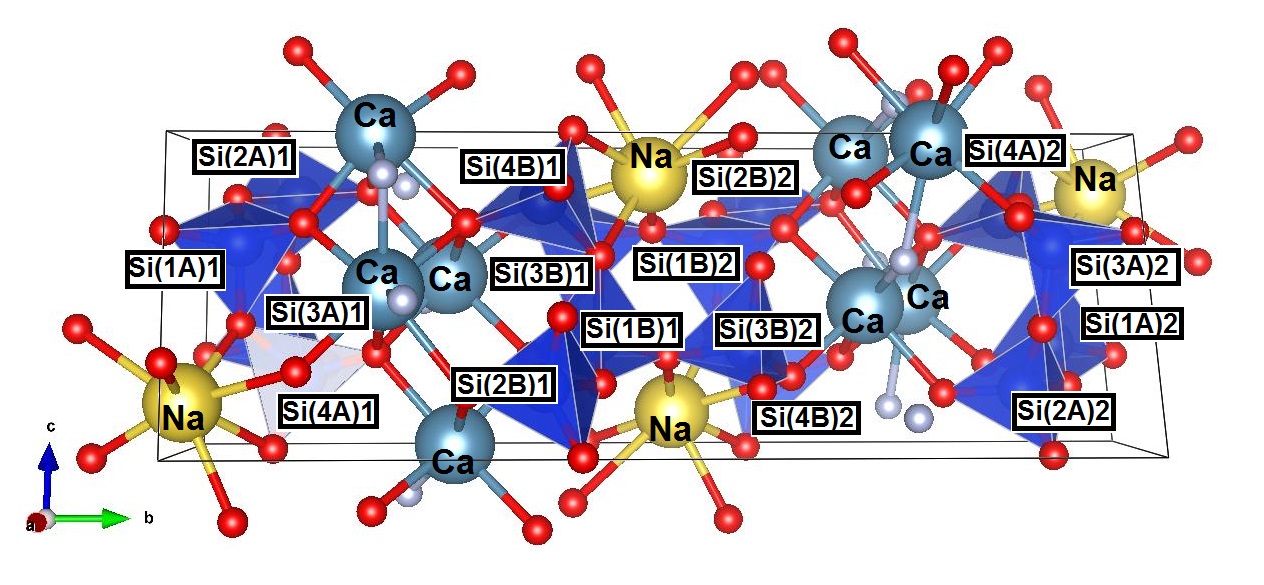 | | | | | | | | | | | | | | | | | | |
| --- | --- | --- | --- | --- | --- | --- | --- | --- | --- | --- | --- | --- | --- | --- | --- | --- | --- | --- |
| Peak position, cm^-1^ | | Int. | **SiO_4_ tetrahedra** | | | | | | | | | | | | | | | |
| Experimental | Calculated | calc. | Si(1A)  1 | Si(1A)  2 | Si(1B)  1 | Si(1B)  2 | Si(2A)  1 | Si(2A)2 | Si(2B)1 | Si(2B)2 | Si(3A)1 | Si(3A)2 | Si(3B)1 | Si(3B)2 | Si(4A)  1 | Si(4A)  2 | Si(4B)  1 | Si(4B)  2 |
| 534 | 509 | 0.49 | B | B | B | B | B | B | B | B | B | B | B | B | B | B | B | B |
| 538 | 511 | 0.001 | B | B | B | B | B | B | B | B | B | B | B | B | B | B | B | B |
| 586 | 562 | 0.93 | AS | AS | -- | -- | AS | AS | B | B | SS | SS | -- | -- | AS | AS | B | B |
| 609 | 573 | 1.09 | -- | -- | AS | AS | -- | -- | SS | SS | -- | -- | SS | SS | B | B | SS | SS |
| 646 | 627 | 0.31 | -- | -- | SS | SS | -- | -- | SS | SS | -- | -- | AS | AS | -- | -- | SS | SS |
| 653 | 636 | 0.12 | SS | SS | -- | -- | SS | SS | -- | -- | AS | AS | -- | -- | SS | SS | -- | -- |
| 681 | 664 | 0.42 | -- | -- | AS | AS | -- | -- | SS | SS | -- | -- | AS | AS | -- | -- | SS | SS |
| 687 | 665 | 0.35 | AS | AS | -- | -- | SS | SS | -- | -- | AS | AS | -- | -- | SS | SS | -- | -- |
| 708 | 690 | 0.07 | -- | -- | SS | SS | -- | -- | AS | AS | -- | -- | SS | SS | -- | -- | AS | AS |
| 712 | 698 | 0.04 | SS | SS | -- | -- | AS | AS | -- | -- | SS | SS | -- | -- | AS | AS | -- | -- |
| 772 | 757 | 0.02 | -- | -- | AS | AS | -- | -- | AS | AS | -- | -- | AS | AS | -- | -- | AS | AS |
| 778 | 761 | 0.45 | AS | AS | AS | AS | AS | AS | B | B | AS | AS | AS | AS | AS | AS | AS | AS |
| 788 | 764 | 0.03 | AS | AS | -- | -- | AS | AS | -- | -- | AS | AS | -- | -- | AS | AS | -- | -- |
| 790 | 772 | 0.07 | AS | AS | AS | AS | B | B | AS | AS | AS | AS | AS | AS | B | B | AS | AS |
| 952 | 937 | 3.01 | -- | -- | AS | AS | -- | -- | AS | AS | -- | -- | -- | -- | -- | -- | AS | AS |
| 961 | 945 | 2.93 | AS | AS | -- | -- | AS | AS | -- | -- | -- | -- | -- | -- | AS | AS | -- | -- |
| 997 | 975 | 3.03 | SS | SS | AS | AS | AS | AS | AS | AS | AS | AS | AS | AS | AS | AS | AS | AS |
| 1003 | 987 | 5.35 | AS | AS | AS | AS | AS | AS | AS | AS | AS | AS | AS | AS | AS | AS | AS | AS |
| 1016 | 990 | 0.51 | -- | -- | AS | AS | -- | -- | AS | AS | AS | AS | AS | AS | B | B | AS | AS |
| 1021 | 993 | 1.61 | AS | AS | -- | -- | AS | AS | -- | -- | AS | AS | -- | -- | AS | AS | -- | -- |
| 1026 | 999 | 1.44 | -- | -- | AS | AS | -- | -- | AS | AS | -- | -- | AS | AS | -- | -- | AS | AS |
| 1039 | 1011 | 0.97 | AS | AS | AS | AS | AS | AS | AS | AS | AS | AS | AS | AS | AS | AS | AS | AS |
| 1050-band | 1024,1029,1034,1036 | 0.16-0.35 | AS | AS | -- | -- | AS | AS | -- | -- | AS | AS | AS | AS | AS | AS | -- | -- |
| 1050-band | 1024,1029,1034,1036 | 0.16-0.35 | AS | AS | AS | AS | AS | AS | AS | AS | AS | AS | AS | AS | AS | AS | AS | AS |
| 1050-band | 1024,1029,1034,1036 | 0.16-0.35 | SS | SS | AS | AS | AS | AS | AS | AS | AS | AS | AS | AS | AS | AS | AS | AS |
| 1050-band | 1024,1029,1034,1036 | 0.16-0.35 | AS | AS | AS | AS | AS | AS | AS | AS | AS | AS | AS | AS | -- | -- | AS | AS |
| 1062 | 1044 | 5.02 | AS | AS | AS | AS | AS | AS | AS | AS | AS | AS | AS | AS | AS | AS | AS | AS |
| 1090 | 1082 | 0.51 | AS | AS | AS | AS | AS | AS | AS | AS | AS | AS | AS | AS | AS | AS | AS | AS |
| 1137 | 1103 | 1.89 | AS | AS | AS | AS | AS | AS | AS | AS | AS | AS | AS | AS | -- | -- | AS | AS |

**Table S9.** Crystallographic coordinates of the model C1 of agrellite.

Position occupancies are taken as 1.

| Site | *x/a* | *y/b* | *z/c* |
| --- | --- | --- | --- |
| Ca(1A)1 | 0.9926 | 0.7857 | 0.9805 |
| Ca(1A)2 | 0.9931 | 0.2132 | 0.9922 |
| Ca(1B)1 | 0.4527 | 0.7176 | 0.9626 |
| Ca(1B)2 | 0.5357 | 0.2817 | 0.0233 |
| Ca(2A)1 | 0.5730 | 0.2779 | 0.5558 |
| Ca(2A)2 | 0.4492 | 0.7218 | 0.4580 |
| Ca(2B)1 | 0.9890 | 0.2199 | 0.5095 |
| Ca(2B)2 | 0.0024 | 0.7809 | 0.4809 |
| Na(A)1 | 0.7544 | 0.0110 | 0.1613 |
| Na(A)2 | 0.2396 | 0.9898 | 0.8312 |
| Na(B)1 | 0.7465 | 0.4941 | 0.8756 |
| Na(B)2 | 0.2565 | 0.5102 | 0.1139 |
| Si(1A)1 | 0.7842 | 0.0676 | 0.6545 |
| Si(1A)2 | 0.2025 | 0.9321 | 0.3305 |
| Si(1B)1 | 0.7009 | 0.4331 | 0.3558 |
| Si(1B)2 | 0.3108 | 0.5701 | 0.6430 |
| Si(2A)1 | 0.5184 | 0.1190 | 0.8032 |
| Si(2A)2 | 0.4780 | 0.8808 | 0.1887 |
| Si(2B)1 | 0.9776 | 0.3798 | 0.2117 |
| Si(2B)2 | 0.0215 | 0.6216 | 0.7788 |
| Si(3A)1 | 0.1604 | 0.0911 | 0.3434 |
| Si(3A)2 | 0.8346 | 0.9101 | 0.6459 |
| Si(3B)1 | 0.3372 | 0.4116 | 0.6767 |
| Si(3B)2 | 0.6662 | 0.5904 | 0.3258 |
| Si(4A)1 | 0.5035 | 0.1243 | 0.2373 |
| Si(4A)2 | 0.4867 | 0.8774 | 0.7506 |
| Si(4B)1 | 0.9936 | 0.3832 | 0.7845 |
| Si(4B)2 | 0.0187 | 0.6191 | 0.2214 |
| F(A)1 | 0.2438 | 0.2266 | 0.8559 |
| F(A)2 | 0.7586 | 0.7602 | 0.1087 |
| F(B)1 | 0.7598 | 0.7571 | 0.6008 |
| F(B)2 | 0.2626 | 0.2457 | 0.4758 |
| O(1A)1 | 0.6236 | 0.0631 | 0.3997 |
| O(1A)2 | 0.3579 | 0.9368 | 0.5872 |
| O(1B)1 | 0.8530 | 0.4392 | 0.6128 |
| O(1B)2 | 0.1668 | 0.5631 | 0.3822 |
| O(2A)1 | 0.9012 | 0.9960 | 0.6912 |
| O(2A)2 | 0.0913 | 0.0055 | 0.2898 |
| O(2B)1 | 0.4213 | 0.4964 | 0.7077 |
| O(2B)2 | 0.5838 | 0.5054 | 0.2979 |
| O(3A)1 | 0.6632 | 0.0598 | 0.7982 |
| O(3A)2 | 0.3318 | 0.9388 | 0.1956 |
| O(3B)1 | 0.8250 | 0.4360 | 0.2186 |
| O(3B)2 | 0.1703 | 0.5663 | 0.7649 |
| O(4A)1 | 0.9229 | 0.1378 | 0.7142 |
| O(4A)2 | 0.0586 | 0.8628 | 0.2640 |
| O(4B)1 | 0.5572 | 0.3630 | 0.2968 |
| O(4B)2 | 0.4551 | 0.6396 | 0.7092 |
| O(5A)1 | 0.6166 | 0.1980 | 0.8252 |
| O(5A)2 | 0.3909 | 0.8007 | 0.1739 |
| O(5B)1 | 0.8853 | 0.2999 | 0.1918 |
| O(5B)2 | 0.1080 | 0.7021 | 0.8039 |
| O(6A)1 | 0.4837 | 0.0926 | 0.0086 |
| O(6A)2 | 0.5099 | 0.9081 | -0.0180 |
| O(6B)1 | -0.0014 | 0.4158 | 0.0056 |
| O(6B)2 | 0.9940 | 0.5915 | -0.0139 |
| O(7A)1 | 0.3046 | 0.0989 | 0.6014 |
| O(7A)2 | 0.6956 | 0.8998 | 0.3865 |
| O(7B)1 | 0.1811 | 0.4005 | 0.4260 |
| O(7B)2 | 0.8065 | 0.6045 | 0.5813 |
| O(8A)1 | 0.0293 | 0.8707 | 0.7336 |
| O(8A)2 | 0.9692 | 0.1324 | 0.2598 |
| O(8B)1 | 0.5178 | 0.3648 | 0.7599 |
| O(8B)2 | 0.4849 | 0.6368 | 0.2309 |
| O(9A)1 | 0.2817 | 0.1182 | 0.2111 |
| O(9A)2 | 0.7076 | 0.8865 | 0.7743 |
| O(9B)1 | 0.2172 | 0.4007 | 0.8208 |
| O(9B)2 | 0.8015 | 0.5999 | 0.2005 |
| O(10A)1 | 0.6071 | 0.2028 | 0.3080 |
| O(10A)2 | 0.3866 | 0.7984 | 0.6838 |
| O(10B)1 | 0.9046 | 0.3030 | 0.7152 |
| O(10B)2 | 0.1038 | 0.6997 | 0.2841 |
| O_W1_ | 0.2203 | 0.3087 | 0.1584 |
| H1 | 0.3420 | 0.3419 | 0.1993 |
| H2 | 0.2318 | 0.2887 | 0.2995 |

**Table S10.** Crystallographic coordinates of the model C2 of agrellite.

***Position occupancies are taken as 1.***

| Site | *x/a* | *y/b* | *z/c* |
| --- | --- | --- | --- |
| Ca(1A)1 | 0.0040 | 0.7842 | 0.0255 |
| Ca(1A)2 | 0.0062 | 0.2057 | 0.9599 |
| Ca(1B)1 | 0.4598 | 0.7126 | 0.9884 |
| Ca(1B)2 | 0.5241 | 0.2828 | 0.9916 |
| Ca(2A)1 | 0.5588 | 0.2708 | 0.5275 |
| Ca(2A)2 | 0.4431 | 0.7202 | 0.4852 |
| Ca(2B)1 | 0.9566 | 0.2227 | 0.4492 |
| Ca(2B)2 | 0.9977 | 0.7810 | 0.5169 |
| Na(A)1 | 0.7591 | 0.0127 | 0.1112 |
| Na(A)2 | 0.2445 | 0.9871 | 0.9250 |
| Na(B)1 | 0.7355 | 0.4861 | 0.7910 |
| Na(B)2 | 0.2511 | 0.5166 | 0.1682 |
| Si(1A)1 | 0.7640 | 0.0638 | 0.6069 |
| Si(1A)2 | 0.2129 | 0.9298 | 0.3695 |
| Si(1B)1 | 0.6872 | 0.4350 | 0.3205 |
| Si(1B)2 | 0.2806 | 0.5682 | 0.6433 |
| Si(2A)1 | 0.5363 | 0.1267 | 0.7798 |
| Si(2A)2 | 0.4961 | 0.8705 | 0.2314 |
| Si(2B)1 | 0.9586 | 0.3857 | 0.1711 |
| Si(2B)2 | 0.0227 | 0.6276 | 0.8029 |
| Si(3A)1 | 0.1742 | 0.0910 | 0.3375 |
| Si(3A)2 | 0.8324 | 0.9051 | 0.6748 |
| Si(3B)1 | 0.3263 | 0.4057 | 0.6223 |
| Si(3B)2 | 0.6646 | 0.5932 | 0.3579 |
| Si(4A)1 | 0.4847 | 0.1219 | 0.1901 |
| Si(4A)2 | 0.4790 | 0.8792 | 0.7838 |
| Si(4B)1 | 0.9896 | 0.3718 | 0.7444 |
| Si(4B)2 | 0.9873 | 0.6197 | 0.2260 |
| F(A)1 | 0.2481 | 0.2122 | 0.8395 |
| F(A)2 | 0.7563 | 0.7615 | 0.1563 |
| F(B)1 | 0.7550 | 0.7600 | 0.6812 |
| F(B)2 | 0.2474 | 0.2722 | 0.5020 |
| O(1A)1 | 0.6127 | 0.0599 | 0.3514 |
| O(1A)2 | 0.3357 | 0.9385 | 0.6339 |
| O(1B)1 | 0.8805 | 0.4260 | 0.5593 |
| O(1B)2 | 0.1263 | 0.5618 | 0.3877 |
| O(2A)1 | 0.8804 | 0.9914 | 0.6576 |
| O(2A)2 | 0.1097 | 0.0033 | 0.2747 |
| O(2B)1 | 0.3902 | 0.4937 | 0.6882 |
| O(2B)2 | 0.6402 | 0.5099 | 0.4141 |
| O(3A)1 | 0.6649 | 0.0619 | 0.7692 |
| O(3A)2 | 0.3758 | 0.9297 | 0.2794 |
| O(3B)1 | 0.8043 | 0.4428 | 0.1672 |
| O(3B)2 | 0.1540 | 0.5659 | 0.7820 |
| O(4A)1 | 0.9153 | 0.1320 | 0.6625 |
| O(4A)2 | 0.0644 | 0.8618 | 0.3012 |
| O(4B)1 | 0.5609 | 0.3597 | 0.2849 |
| O(4B)2 | 0.4333 | 0.6354 | 0.7152 |
| O(5A)1 | 0.6527 | 0.2034 | 0.8404 |
| O(5A)2 | 0.3901 | 0.7929 | 0.1978 |
| O(5B)1 | 0.8774 | 0.3060 | 0.1724 |
| O(5B)2 | 0.1254 | 0.7058 | 0.8476 |
| O(6A)1 | 0.4812 | 0.0938 | 0.9644 |
| O(6A)2 | 0.5165 | 0.9051 | 0.0253 |
| O(6B)1 | 0.9971 | 0.4099 | 0.9626 |
| O(6B)2 | 0.9690 | 0.5945 | 0.9902 |
| O(7A)1 | 0.3772 | 0.1322 | 0.5292 |
| O(7A)2 | 0.7181 | 0.8757 | 0.4250 |
| O(7B)1 | 0.1753 | 0.4094 | 0.3701 |
| O(7B)2 | 0.8211 | 0.6298 | 0.5861 |
| O(8A)1 | 0.0356 | 0.8720 | 0.7967 |
| O(8A)2 | 0.9857 | 0.1355 | 0.2367 |
| O(8B)1 | 0.5285 | 0.3714 | 0.7138 |
| O(8B)2 | 0.4666 | 0.6309 | 0.2583 |
| O(9A)1 | 0.2594 | 0.1045 | 0.1467 |
| O(9A)2 | 0.6943 | 0.8982 | 0.7958 |
| O(9B)1 | 0.2168 | 0.3774 | 0.7724 |
| O(9B)2 | 0.7658 | 0.6019 | 0.1948 |
| O(10A)1 | 0.5919 | 0.1997 | 0.2721 |
| O(10A)2 | 0.3887 | 0.7992 | 0.7131 |
| O(10B)1 | 0.8957 | 0.2920 | 0.6920 |
| O(10B)2 | 0.0878 | 0.6981 | 0.3067 |
| O_W1_ | 0.2373 | 0.2959 | 0.1535 |
| H1 | 0.3521 | 0.3336 | 0.1943 |
| H2 | 0.2178 | 0.2919 | 0.2882 |
| O_W2_ | 0.1427 | 0.0639 | 0.5955 |
| H3 | 0.0507 | 0.0216 | 0.5674 |
| H4 | 0.0760 | 0.1012 | 0.6471 |
| O_W3_ | 0.4503 | 0.4642 | 0.0650 |
| H5 | 0.4696 | 0.4894 | 0.9491 |
| H6 | 0.3583 | 0.4224 | 0.9805 |

**Table S11.** Crystallographic coordinates of the model C3 of agrellite.

***Position occupancies are taken as 1.***

| Site | *x/a* | *y/b* | *z/c* |
| --- | --- | --- | --- |
| Ca(1A)1 | 0.0440 | 0.7877 | 0.0462 |
| Ca(1A)2 | -0.0038 | 0.2072 | 0.9625 |
| Ca(1B)1 | 0.4866 | 0.7154 | 0.0233 |
| Ca(1B)2 | 0.5136 | 0.2817 | 0.9900 |
| Ca(2A)1 | 0.5552 | 0.2705 | 0.5291 |
| Ca(2A)2 | 0.4934 | 0.7210 | 0.5358 |
| Ca(2B)1 | 0.9487 | 0.2225 | 0.4469 |
| Ca(2B)2 | 0.0427 | 0.7868 | 0.5510 |
| Na(A)1 | 0.7527 | 0.0161 | 0.1183 |
| Na(A)2 | 0.2355 | 0.9918 | 0.9205 |
| Na(B)1 | 0.7279 | 0.4733 | 0.7546 |
| Na(B)2 | 0.2442 | 0.5182 | 0.1780 |
| Si(1A)1 | 0.7597 | 0.0656 | 0.6137 |
| Si(1A)2 | 0.1906 | 0.9320 | 0.3615 |
| Si(1B)1 | 0.6882 | 0.4331 | 0.3143 |
| Si(1B)2 | 0.2562 | 0.5657 | 0.6588 |
| Si(2A)1 | 0.5277 | 0.1262 | 0.7818 |
| Si(2A)2 | 0.4828 | 0.8776 | 0.2274 |
| Si(2B)1 | 0.9570 | 0.3799 | 0.1640 |
| Si(2B)2 | 0.0100 | 0.6318 | 0.8207 |
| Si(3A)1 | 0.1664 | 0.0923 | 0.3376 |
| Si(3A)2 | 0.8247 | 0.9098 | 0.6747 |
| Si(3B)1 | 0.3150 | 0.4034 | 0.6185 |
| Si(3B)2 | 0.6723 | 0.5994 | 0.3874 |
| Si(4A)1 | 0.4794 | 0.1207 | 0.1942 |
| Si(4A)2 | 0.4733 | 0.8834 | 0.7848 |
| Si(4B)1 | 0.9899 | 0.3684 | 0.7426 |
| Si(4B)2 | 0.0153 | 0.6380 | 0.2695 |
| F(A)1 | 0.2443 | 0.2084 | 0.8408 |
| F(A)2 | 0.7368 | 0.7385 | -0.0832 |
| F(B)1 | 0.7337 | 0.7495 | 0.4326 |
| F(B)2 | 0.2438 | 0.2673 | 0.5010 |
| O(1A)1 | 0.6096 | 0.0586 | 0.3575 |
| O(1A)2 | 0.3212 | 0.9386 | 0.6254 |
| O(1B)1 | 0.8818 | 0.4219 | 0.5554 |
| O(1B)2 | 0.1164 | 0.5701 | 0.4039 |
| O(2A)1 | 0.8872 | 0.9953 | 0.6782 |
| O(2A)2 | 0.0887 | 0.0055 | 0.2764 |
| O(2B)1 | 0.3420 | 0.4899 | 0.6663 |
| O(2B)2 | 0.6735 | 0.5138 | 0.4070 |
| O(3A)1 | 0.6578 | 0.0614 | 0.7729 |
| O(3A)2 | 0.3435 | 0.9325 | 0.2581 |
| O(3B)1 | 0.8066 | 0.4363 | 0.1652 |
| O(3B)2 | 0.1226 | 0.5653 | 0.7884 |
| O(4A)1 | 0.9060 | 0.1345 | 0.6653 |
| O(4A)2 | 0.0390 | 0.8645 | 0.2926 |
| O(4B)1 | 0.5616 | 0.3582 | 0.2886 |
| O(4B)2 | 0.4196 | 0.6315 | 0.7431 |
| O(5A)1 | 0.6430 | 0.2027 | 0.8405 |
| O(5A)2 | 0.3871 | 0.7984 | 0.1954 |
| O(5B)1 | 0.8736 | 0.3002 | 0.1604 |
| O(5B)2 | 0.1205 | 0.7077 | 0.8351 |
| O(6A)1 | 0.4761 | 0.0924 | 0.9684 |
| O(6A)2 | 0.5065 | 0.9118 | 0.0221 |
| O(6B)1 | 0.9958 | 0.4066 | 0.9584 |
| O(6B)2 | 0.9947 | 0.6114 | 0.0388 |
| O(7A)1 | 0.3730 | 0.1305 | 0.5288 |
| O(7A)2 | 0.7007 | 0.8897 | 0.4210 |
| O(7B)1 | 0.1718 | 0.4021 | 0.3629 |
| O(7B)2 | 0.7835 | 0.6257 | 0.6409 |
| O(8A)1 | 0.0172 | 0.8698 | 0.7881 |
| O(8A)2 | 0.9789 | 0.1369 | 0.2356 |
| O(8B)1 | 0.5208 | 0.3718 | 0.7077 |
| O(8B)2 | 0.4625 | 0.6287 | 0.2710 |
| O(9A)1 | 0.2536 | 0.1021 | 0.1487 |
| O(9A)2 | 0.6867 | 0.9006 | 0.7944 |
| O(9B)1 | 0.2165 | 0.3711 | 0.7743 |
| O(9B)2 | 0.8014 | 0.6325 | 0.2663 |
| O(10A)1 | 0.5863 | 0.1983 | 0.2741 |
| O(10A)2 | 0.3916 | 0.8024 | 0.7270 |
| O(10B)1 | 0.8905 | 0.2893 | 0.6913 |
| O(10B)2 | 0.1305 | 0.7137 | 0.3472 |
| O_W1_ | 0.2402 | 0.2931 | 0.1558 |
| H1 | 0.3507 | 0.3328 | 0.1964 |
| H2 | 0.2191 | 0.2884 | 0.2897 |
| O_W2_ | 0.1363 | 0.0675 | 0.5994 |
| H3 | 0.0422 | 0.0259 | 0.5699 |
| H4 | 0.0730 | 0.1049 | 0.6541 |
| O_W3_ | 0.4565 | 0.4641 | 0.0754 |
| H5 | 0.5194 | 0.4970 | -0.0034 |
| H6 | 0.3684 | 0.4252 | 0.9752 |
| O_W4_ | 0.6487 | 0.5414 | -0.0589 |
| H7 | 0.7655 | 0.5543 | 0.0722 |
| H8 | 0.5875 | 0.5869 | -0.1287 |

**Table S12.** Crystallographic coordinates of the model C4 of agrellite.

***Position occupancies are taken as 1.***

| Site | *x/a* | *y/b* | *z/c* |
| --- | --- | --- | --- |
| Ca(1A)1 | 0.0282 | 0.7880 | 0.0384 |
| Ca(1A)2 | 0.0137 | 0.2023 | 0.9810 |
| Ca(1B)1 | 0.4920 | 0.7122 | 0.0163 |
| Ca(1B)2 | 0.5470 | 0.2928 | 0.0000 |
| Ca(2A)1 | 0.5842 | 0.2794 | 0.5381 |
| Ca(2A)2 | 0.4849 | 0.7209 | 0.5308 |
| Ca(2B)1 | 0.9647 | 0.2208 | 0.4410 |
| Ca(2B)2 | 0.0305 | 0.7897 | 0.5528 |
| Na(A)1 | 0.7380 | 0.0139 | 0.0998 |
| Na(A)2 | 0.2075 | 0.9925 | 0.9381 |
| Na(B)1 | 0.7784 | 0.4830 | 0.7875 |
| Na(B)2 | 0.3632 | 0.4747 | 0.0485 |
| Si(1A)1 | 0.7956 | 0.0747 | 0.6063 |
| Si(1A)2 | 0.1303 | 0.9250 | 0.3260 |
| Si(1B)1 | 0.7464 | 0.4358 | 0.3491 |
| Si(1B)2 | 0.2512 | 0.5687 | 0.6690 |
| Si(2A)1 | 0.5526 | 0.1370 | 0.7790 |
| Si(2A)2 | 0.4459 | 0.8716 | 0.2045 |
| Si(2B)1 | 0.9931 | 0.3786 | 0.1897 |
| Si(2B)2 | 0.0147 | 0.6306 | 0.8362 |
| Si(3A)1 | 0.1966 | 0.0843 | 0.3528 |
| Si(3A)2 | 0.7886 | 0.9148 | 0.6592 |
| Si(3B)1 | 0.3438 | 0.4070 | 0.6483 |
| Si(3B)2 | 0.6755 | 0.5999 | 0.3827 |
| Si(4A)1 | 0.4794 | 0.1370 | 0.1621 |
| Si(4A)2 | 0.4434 | 0.8826 | 0.7713 |
| Si(4B)1 | 0.0277 | 0.3658 | 0.7679 |
| Si(4B)2 | -0.0002 | 0.6430 | 0.2723 |
| F(A)1 | 0.3249 | 0.1974 | 0.9499 |
| F(A)2 | 0.7339 | 0.7340 | 0.9061 |
| F(B)1 | 0.7348 | 0.7511 | 0.4652 |
| F(B)2 | 0.2789 | 0.2670 | 0.5688 |
| O(1A)1 | 0.6361 | 0.0722 | 0.3530 |
| O(1A)2 | 0.2515 | 0.9192 | 0.5940 |
| O(1B)1 | 0.9120 | 0.4242 | 0.5938 |
| O(1B)2 | 0.0744 | 0.5769 | 0.4313 |
| O(2A)1 | 0.8854 | 0.9965 | 0.6598 |
| O(2A)2 | 0.0521 | 0.0029 | 0.2425 |
| O(2B)1 | 0.3188 | 0.4896 | 0.6843 |
| O(2B)2 | 0.6687 | 0.5129 | 0.3571 |
| O(3A)1 | 0.6999 | 0.0785 | 0.7721 |
| O(3A)2 | 0.2593 | 0.9074 | 0.2003 |
| O(3B)1 | 0.8597 | 0.4407 | 0.1987 |
| O(3B)2 | 0.1486 | 0.5675 | 0.8315 |
| O(4A)1 | 0.9700 | 0.1352 | 0.6778 |
| O(4A)2 | 0.9609 | 0.8625 | 0.2569 |
| O(4B)1 | 0.5726 | 0.3745 | 0.2670 |
| O(4B)2 | 0.4209 | 0.6307 | 0.7335 |
| O(5A)1 | 0.6695 | 0.2133 | 0.8453 |
| O(5A)2 | 0.3870 | 0.7891 | 0.1829 |
| O(5B)1 | 0.8904 | 0.3014 | 0.1747 |
| O(5B)2 | 0.1090 | 0.7076 | 0.8310 |
| O(6A)1 | 0.5103 | 0.0982 | 0.9668 |
| O(6A)2 | 0.4659 | 0.9121 | 0.0038 |
| O(6B)1 | 0.0347 | 0.3997 | 0.9858 |
| O(6B)2 | 0.9839 | 0.6130 | 0.0475 |
| O(7A)1 | 0.3821 | 0.1417 | 0.5380 |
| O(7A)2 | 0.6462 | 0.8986 | 0.4075 |
| O(7B)1 | 0.1994 | 0.3893 | 0.3995 |
| O(7B)2 | 0.7956 | 0.6117 | 0.6423 |
| O(8A)1 | 0.9641 | 0.8674 | 0.7708 |
| O(8A)2 | 0.0162 | 0.1358 | 0.2563 |
| O(8B)1 | 0.5558 | 0.3809 | 0.7299 |
| O(8B)2 | 0.4673 | 0.6306 | 0.2831 |
| O(9A)1 | 0.2772 | 0.0986 | 0.1561 |
| O(9A)2 | 0.6440 | 0.9202 | 0.7743 |
| O(9B)1 | 0.2547 | 0.3705 | 0.8102 |
| O(9B)2 | 0.7796 | 0.6428 | 0.2500 |
| O(10A)1 | 0.6202 | 0.2063 | 0.2934 |
| O(10A)2 | 0.3997 | 0.7992 | 0.7332 |
| O(10B)1 | 0.9252 | 0.2877 | 0.7013 |
| O(10B)2 | 0.1229 | 0.7173 | 0.3483 |
| O_W1_ | 0.2734 | 0.2825 | 0.2136 |
| H1 | 0.3314 | 0.3307 | 0.2076 |
| H2 | 0.2481 | 0.2825 | 0.3465 |
| O_W2_ | 0.1739 | 0.0573 | 0.6074 |
| H3 | 0.0727 | 0.0176 | 0.5715 |
| H4 | 0.1117 | 0.0985 | 0.6541 |
| O_W4_ | 0.6389 | 0.5330 | 0.9553 |
| H7 | 0.7655 | 0.5454 | 0.0741 |
| H8 | 0.5920 | 0.5784 | 0.8812 |
| O_W5_ | 0.3947 | 0.0132 | 0.4525 |
| H9 | 0.4884 | 0.0302 | 0.3899 |
| H10 | 0.4761 | 0.0112 | 0.6077 |
| O_W6_ | 0.2783 | 0.5143 | 0.2716 |
| H11 | 0.1556 | 0.5167 | 0.2771 |
| H12 | 0.3272 | 0.5652 | 0.2679 |

**Table S13.** Mean interatomic distances (Å), bond-valence sum (BVS), tetrahedral volumes (Å^3^) and distortion parameters for Si-tetrahedra in the simulated H_2_O-containing agrellite structural models. Sp. gr. *P*1.

| <Si-O> (Å) | | | | | | | | | |
| --- | --- | --- | --- | --- | --- | --- | --- | --- | --- |
| Site | C1 | C2 | C3 | C4 |  | C1 | C2 | C3 | C4 |
| Si(1A)1 | 1.63 | 1.63 | 1.63 | 1.63 | Si(3A)1 | 1.64 | (*) | (*) | (*) |
| Si(1A)2 | 1.63 | 1.63 | 1.63 | 1.65 | Si(3A)2 | 1.64 | 1.63 | 1.62 | 1.63 |
| Si(1B)1 | 1.63 | 1.64 | (*) | 1.63 | Si(3B)1 | 1.63 | 1.65 | 1.64 | 1.63 |
| Si(1B)2 | 1.64 | 1.64 | 1.63 | 1.62 | Si(3B)2 | 1.63 | 1.63 | 1.64 | 1.64 |
| Si(2A)1 | 1.64 | 1.64 | 1.64 | 1.63 | Si(4A)1 | 1.63 | 1.64 | 1.65 | (*) |
| Si(2A)2 | 1.64 | 1.63 | 1.63 | 1.62 | Si(4A)2 | 1.63 | 1.64 | 1.63 | 1.64 |
| Si(2B)1 | 1.64 | 1.65 | 1.64 | 1.62 | Si(4B)1 | 1.64 | 1.64 | 1.63 | 1.63 |
| Si(2B)2 | 1.63 | 1.63 | 1.63 | 1.63 | Si(4B)2 | 1.63 | 1.64 | 1.62 | 1.62 |
| Bond valence sum (v.u.) | | | | | | | | | |
| Site | C1 | C2 | C3 | C4 |  | C1 | C2 | C3 | C4 |
| Si(1A)1 | 3.94 | 3.95 | 3.95 | 3.92 | Si(3A)1 | 3.90 | (*) | (*) | (*) |
| Si(1A)2 | 3.91 | 3.92 | 3.96 | 3.80 | Si(3A)2 | 3.89 | 3.91 | 4.01 | 3.95 |
| Si(1B)1 | 3.94 | 3.54 | (*) | 3.94 | Si(3B)1 | 3.90 | 3.74 | 3.87 | 3.95 |
| Si(1B)2 | 3.89 | 3.88 | 3.97 | 4.02 | Si(3B)2 | 3.92 | 3.95 | 3.90 | 3.89 |
| Si(2A)1 | 3.88 | 3.90 | 3.89 | 3.93 | Si(4A)1 | 3.90 | 3.81 | 3.78 | (*) |
| Si(2A)2 | 3.89 | 3.95 | 3.99 | 4.04 | Si(4A)2 | 3.90 | 3.87 | 3.93 | 3.90 |
| Si(2B)1 | 3.83 | 3.78 | 3.88 | 4.03 | Si(4B)1 | 3.86 | 3.89 | 3.94 | 3.99 |
| Si(2B)2 | 3.93 | 3.95 | 3.96 | 3.97 | Si(4B)2 | 3.90 | 3.87 | 4.04 | 4.04 |
| Tetrahedral volume (Å^3^) | | | | | | | | | |
| Site | C1 | C2 | C3 | C4 |  | C1 | C2 | C3 | C4 |
| Si(1A)1 | 2.22 | 2.21 | 2.21 | 2.21 | Si(3A)1 | 2.23 | (*) | (*) | (*) |
| Si(1A)2 | 2.23 | 2.22 | 2.21 | 2.26 | Si(3A)2 | 2.24 | 2.22 | 2.19 | 2.20 |
| Si(1B)1 | 2.22 | 2.23 | (*) | 2.22 | Si(3B)1 | 2.23 | 2.25 | 2.20 | 2.18 |
| Si(1B)2 | 2.24 | 2.24 | 2.20 | 2.18 | Si(3B)2 | 2.23 | 2.21 | 2.22 | 2.21 |
| Si(2A)1 | 2.21 | 2.16 | 2.15 | 2.17 | Si(4A)1 | 2.21 | 2.23 | 2.24 | (*) |
| Si(2A)2 | 2.21 | 2.19 | 2.17 | 2.14 | Si(4A)2 | 2.21 | 2.22 | 2.21 | 2.21 |
| Si(2B)1 | 2.21 | 2.25 | 2.21 | 2.17 | Si(4B)1 | 2.22 | 2.23 | 2.21 | 2.18 |
| Si(2B)2 | 2.20 | 2.19 | 2.19 | 2.19 | Si(4B)2 | 2.21 | 2.22 | 2.14 | 2.15 |
| Tetrahedral angle variance (TAV) | | | | | | | | | |
| Site | C1 | C2 | C3 | C4 |  | C1 | C2 | C3 | C4 |
| Si(1A)1 | 10.43 | 13.89 | 17.43 | 27.72 | Si(3A)1 | 12.19 | (*) | (*) | (*) |
| Si(1A)2 | 15.03 | 20.64 | 19.34 | 25.96 | Si(3A)2 | 11.04 | 17.12 | 15.77 | 39.74 |
| Si(1B)1 | 7.02 | 165.51 | (*) | 9.90 | Si(3B)1 | 17.09 | 79.76 | 66.88 | 59.27 |
| Si(1B)2 | 15.43 | 15.98 | 20.05 | 24.05 | Si(3B)2 | 12.21 | 15.62 | 24.35 | 40.16 |
| Si(2A)1 | 52.61 | 92.37 | 113.37 | 74.09 | Si(4A)1 | 34.34 | 61.78 | 68.09 | (*) |
| Si(2A)2 | 48.16 | 36.47 | 40.24 | 62.52 | Si(4A)2 | 31.49 | 45.04 | 31.85 | 45.59 |
| Si(2B)1 | 77.13 | 51.22 | 54.35 | 19.57 | Si(4B)1 | 51.04 | 25.69 | 17.99 | 29.83 |
| Si(2B)2 | 38.06 | 35.06 | 30.58 | 39.25 | Si(4B)2 | 38.81 | 40.13 | 55.49 | 48.76 |
| Tetrahedral quadratic elongation (TQE) | | | | | | | | | |
| Site | C1 | C2 | C3 | C4 |  | C1 | C2 | C3 | C4 |
| Si(1A)1 | 1.002 | 1.004 | 1.005 | 1.007 | Si(3A)1 | 1.003 | (*) | (*) | (*) |
| Si(1A)2 | 1.003 | 1.005 | 1.005 | 1.008 | Si(3A)2 | 1.003 | 1.004 | 1.004 | 1.009 |
| Si(1B)1 | 1.002 | 1.053 | (*) | 1.002 | Si(3B)1 | 1.004 | 1.019 | 1.017 | 1.015 |
| Si(1B)2 | 1.004 | 1.004 | 1.005 | 1.006 | Si(3B)2 | 1.003 | 1.004 | 1.006 | 1.010 |
| Si(2A)1 | 1.012 | 1.025 | 1.031 | 1.020 | Si(4A)1 | 1.008 | 1.014 | 1.016 | (*) |
| Si(2A)2 | 1.011 | 1.009 | 1.010 | 1.016 | Si(4A)2 | 1.007 | 1.010 | 1.008 | 1.011 |
| Si(2B)1 | 1.019 | 1.012 | 1.013 | 1.005 | Si(4B)1 | 1.012 | 1.006 | 1.005 | 1.007 |
| Si(2B)2 | 1.009 | 1.009 | 1.008 | 1.009 | Si(4B)2 | 1.009 | 1.009 | 1.014 | 1.011 |
| Bond length distortion (BLD, %) | | | | | | | | | |
| Site | C1 | C2 | C3 | C4 |  | C1 | C2 | C3 | C4 |
| Si(1A)1 | 1.65 | 0.84 | 1.20 | 1.04 | Si(3A)1 | 1.18 | (*) | (*) | (*) |
| Si(1A)2 | 1.60 | 1.53 | 1.31 | 1.39 | Si(3A)2 | 1.16 | 1.17 | 1.00 | 1.93 |
| Si(1B)1 | 1.09 | 1.52 | (*) | 0.84 | Si(3B)1 | 1.49 | 2.58 | 1.65 | 1.87 |
| Si(1B)2 | 1.53 | 1.55 | 0.79 | 1.05 | Si(3B)2 | 1.26 | 1.10 | 1.22 | 1.16 |
| Si(2A)1 | 1.06 | 1.00 | 1.19 | 1.54 | Si(4A)1 | 0.96 | 1.21 | 1.47 | (*) |
| Si(2A)2 | 1.15 | 1.11 | 1.07 | 1.51 | Si(4A)2 | 0.98 | 1.11 | 1.08 | 1.43 |
| Si(2B)1 | 1.33 | 1.62 | 1.29 | 1.21 | Si(4B)1 | 1.12 | 2.08 | 1.82 | 1.07 |
| Si(2B)2 | 1.19 | 1.45 | 1.07 | 1.14 | Si(4B)2 | 1.12 | 1.11 | 1.00 | 1.03 |
| (*) Tetrahedron gains CN more than 4. | | | | | | | | | |

**Table S14.** Coordination numbers (CN), mean interatomic distances (Å) and bond-valence sum (BVS) of Ca- and Na-polyhedra in the crystal structures of the simulated H_2_O-containing agrellite structural models. Sp. gr. *P*1.

| **Ca-polyhedra** | | | | | | | |
| --- | --- | --- | --- | --- | --- | --- | --- |
| Site | CN | <Ca-O,F,Ow> | BVS | Site | CN | <Ca-O,F,Ow > | BVS |
| Model C1 | | | | | | | |
| Ca(1A)1 | 8 | 2.53 | 1.74 | Ca(1B)1 | 6 | 2.34 | 2.03 |
| Ca(1A)2 | 9 | 2.57 | 1.96 | Ca(1B)2 | 7 | 2.46 | 1.96 |
| Ca(2A)1 | 7 | 2.43 | 2.09 | Ca(2B)1 | 7 | 2.41 | 1.96 |
| Ca(2A)2 | 6 | 2.34 | 1.98 | Ca(2B)2 | 7 | 2.40 | 1.73 |
| Model C2 | | | | | | | |
| Ca(1A)1 | 7 | 2.43 | 1.87 | Ca(1B)1 | 7 | 2.45 | 2.06 |
| Ca(1A)2 | 8 | 2.49 | 2.07 | Ca(1B)2 | 9 | 2.60 | 1.91 |
| Ca(2A)1 | 8 | 2.48 | 2.09 | Ca(2B)1 | 7 | 2.41 | 1.99 |
| Ca(2A)2 | 6 | 2.35 | 1.95 | Ca(2B)2 | 7 | 2.51 | 1.53 |
| Model C3 | | | | | | | |
| Ca(1A)1 | 6 | 2.34 | 2.01 | Ca(1B)1 | 8 | 2.52 | 1.83 |
| Ca(1A)2 | 8 | 2.47 | 2.14 | Ca(1B)2 | 9 | 2.59 | 1.91 |
| Ca(2A)1 | 8 | 2.47 | 2.17 | Ca(2B)1 | 7 | 2.40 | 2.05 |
| Ca(2A)2 | 8 | 2.51 | 1.78 | Ca(2B)2 | 6 | 2.34 | 2.03 |
| Model C4 | | | | | | | |
| Ca(1A)1 | 7 | 2.40 | 2.10 | Ca(1B)1 | 8 | 2.50 | 1.94 |
| Ca(1A)2 | 9 | 2.56 | 2.06 | Ca(1B)2 | 9 | 2.55 | 2.03 |
| Ca(2A)1 | 8 | 2.49 | 2.03 | Ca(2B)1 | 6 | 2.31 | 2.15 |
| Ca(2A)2 | 8 | 2.50 | 1.97 | Ca(2B)2 | 7 | 2.43 | 2.06 |
| **Na-polyhedra** | | | | | | | |
| Site | CN | <Na-O,Ow > | BVS | Site | CN | <Na-O,Ow > | BVS |
| Model C1 | | | | Model C2 | | | |
| Na(A)1 | 7 | 2.55 | 0.95 | Na(A)1 | 7 | 2.60 | 0.88 |
| Na(A)2 | 7 | 2.54 | 0.96 | Na(A)2 | 9 | 2.63 | 1.03 |
| Na(B)1 | 8 | 2.61 | 1.08 | Na(B)1 | 7 | 2.52 | 1.00 |
| Na(B)2 | 7 | 2.54 | 1.02 | Na(B)2 | 8 | 2.54 | 1.14 |
| Model C3 | | | | Model C4 | | | |
| Na(A)1 | 7 | 2.56 | 0.98 | Na(A)1 | 7 | 2.53 | 1.07 |
| Na(A)2 | 9 | 2.61 | 1.12 | Na(A)2 | 9 | 2.63 | 1.11 |
| Na(B)1 | 8 | 2.54 | 1.30 | Na(B)1 | 9 | 2.64 | 1.18 |
| Na(B)2 | 8 | 2.61 | 0.98 | Na(B)2 | 8 | 2.48 | 1.35 |
| Model C1 – agrellite phase with 1 H_2_O molecule per unit cell (NaCa_2_Si_4_O_10_F·0.25H_2_O); model C2 – agrellite phase with 3 H_2_O molecules per unit cell (NaCa_2_Si_4_O_10_F·0.75H_2_O); model C3 – agrellite phase with 4 H_2_O molecules per unit cell (NaCa_2_Si_4_O_10_F·1H_2_O); model C4 – agrellite phase with 5 H_2_O molecules per unit cell (NaCa_2_Si_4_O_10_F·1.25H_2_O) | | | | | | | |

**Table S15.** The global instability index (GII, %) calculated for natural agrellite crystal structure and simulated H_2_O-containing agrellite structural models

|  | GII (%)  **Ca** | GII (%)  **Na** | GII (%)  **Si** | GII (%) **O** | GII (%)  **F** | GII (%)  **H** | **GII (%) Total** |
| --- | --- | --- | --- | --- | --- | --- | --- |
| Agrellite | 21.85 | 7.32 | 5.81 | 13.29 | 20.42 | - | **13.60** |
| Model C1 | 13.98 | 5.19 | 10.79 | 12.10 | 18.65 | 8.05 | **12.14** |
| Model C2 | 18.29 | 9.19 | 17.44 | 13.03 | 23.75 | 4.40 | **14.72** |
| Model C3 | 13.19 | 16.22 | 9.08 | 12.19 | 24.42 | 8.35 | **12.56** |
| Model C4 | 7.63 | 20.74 | 9.17 | 14.59 | 16.73 | 8.70 | **13.13** |

**FIGURES**

**
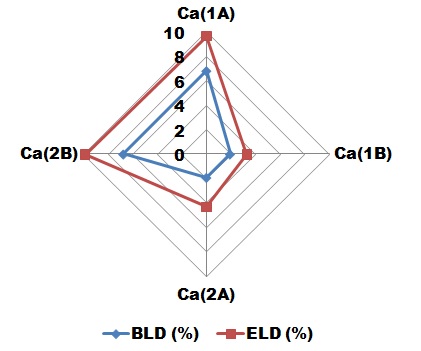
**

**Figure S1**. Bond length distortion (BLD) and edge length distortion (ELD) values, calculated for Ca(1A) and Ca(2B) polyhedra and Ca(1B) and Ca(2A) octahedra of the agrellite crystal structure.

**
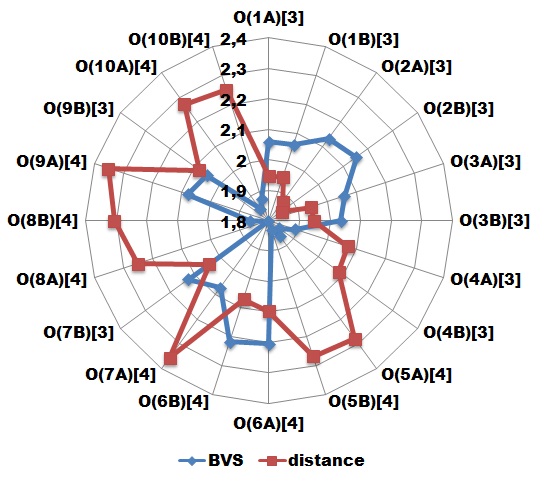
**

**Figure S2**. Mean anion-cation distances (Å) and bond valence sums (vu) for oxygen positions in the crystal structure of agrellite. Coordination numbers are given in square brackets.

**
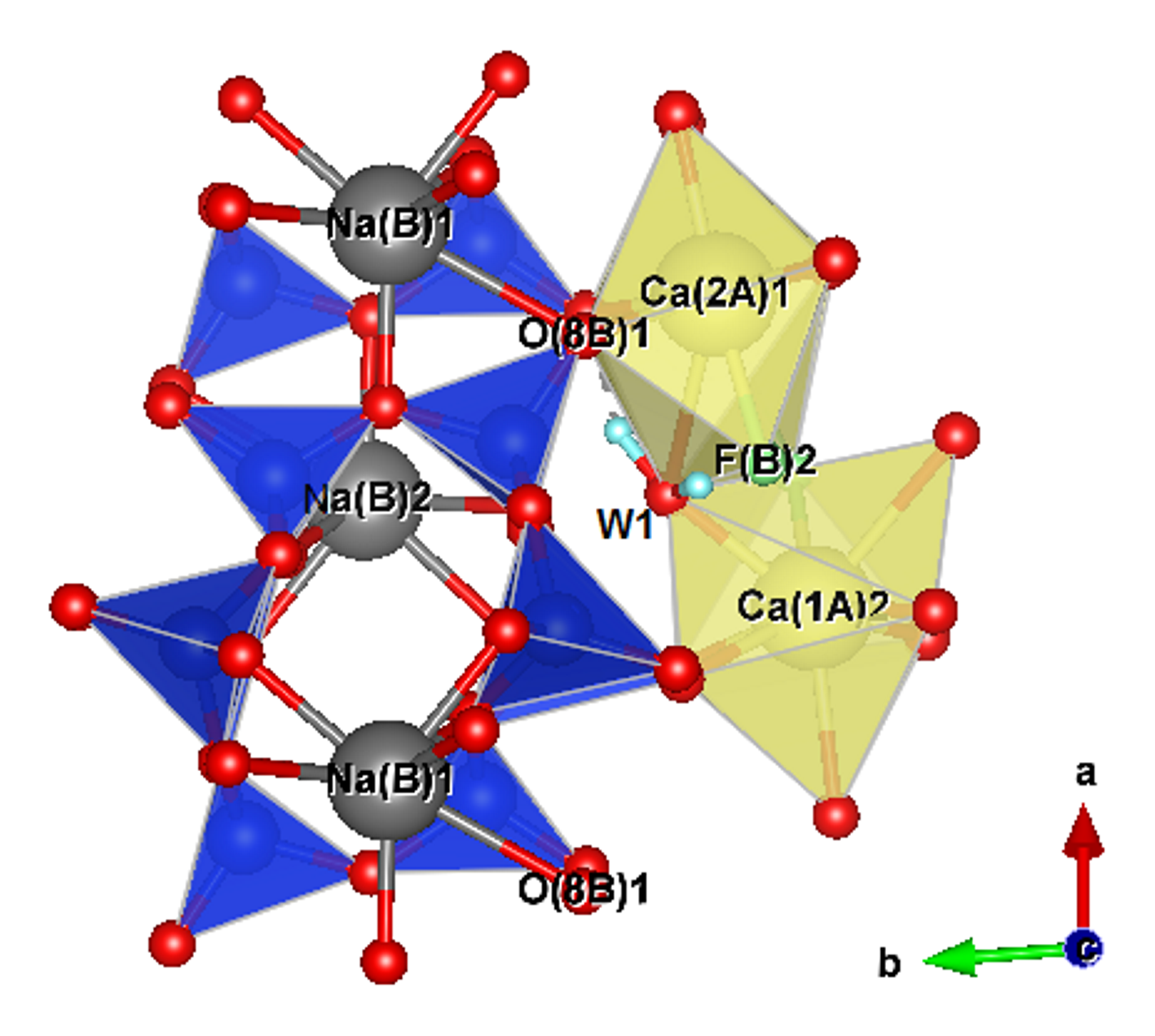
**

**Figure S3**. Position of the H_2_O molecule in the simulated structural model C1 with one H_2_O molecule per unit cell (1.1 wt.%). SiO_4_ tetrahedra and Ca-polyhedra are drawn in blue and yellow; sodium and fluorine are drawn in grey and green; oxygen and hydrogen atoms are red and cyan, respectively.

**
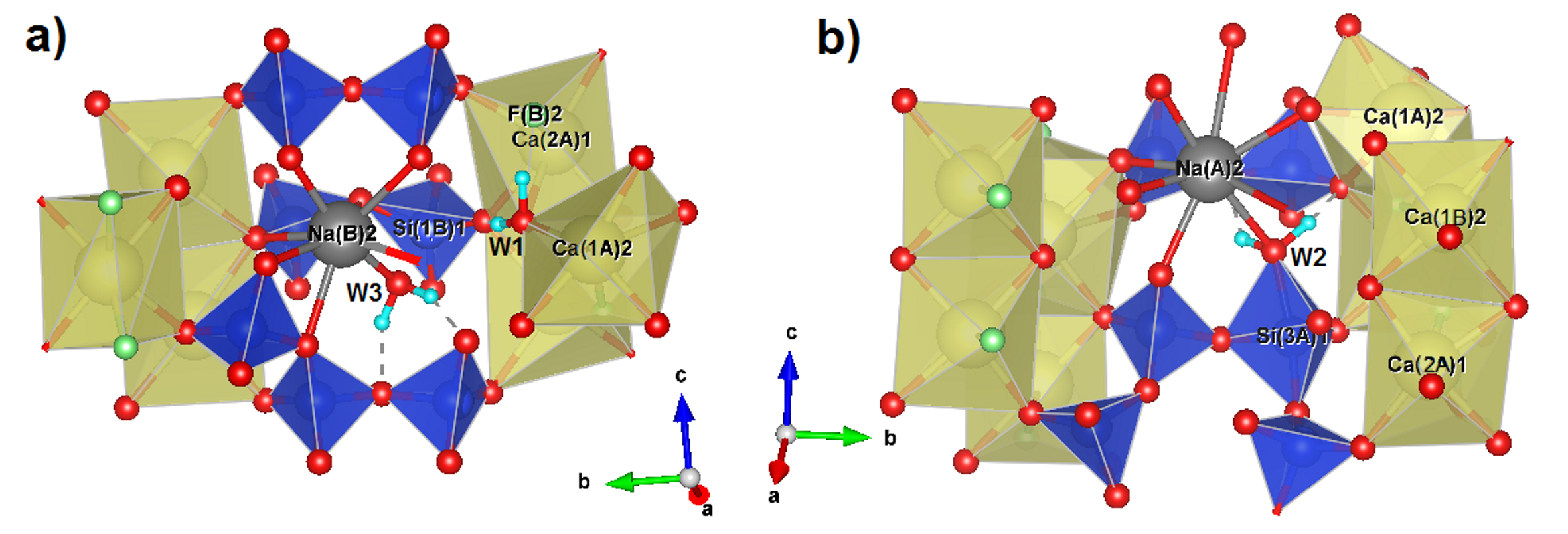
**

**Figure S4**. Positions of the H_2_O molecules in the simulated structural model C2 with three H_2_O molecules per unit cell (3.3 wt.%): *a*) W1 and W3 molecules in the p1 and p3 positions, *b*) W2 molecule in the p2 position. The definitions of colors are the ASme as in Figure S3.

**
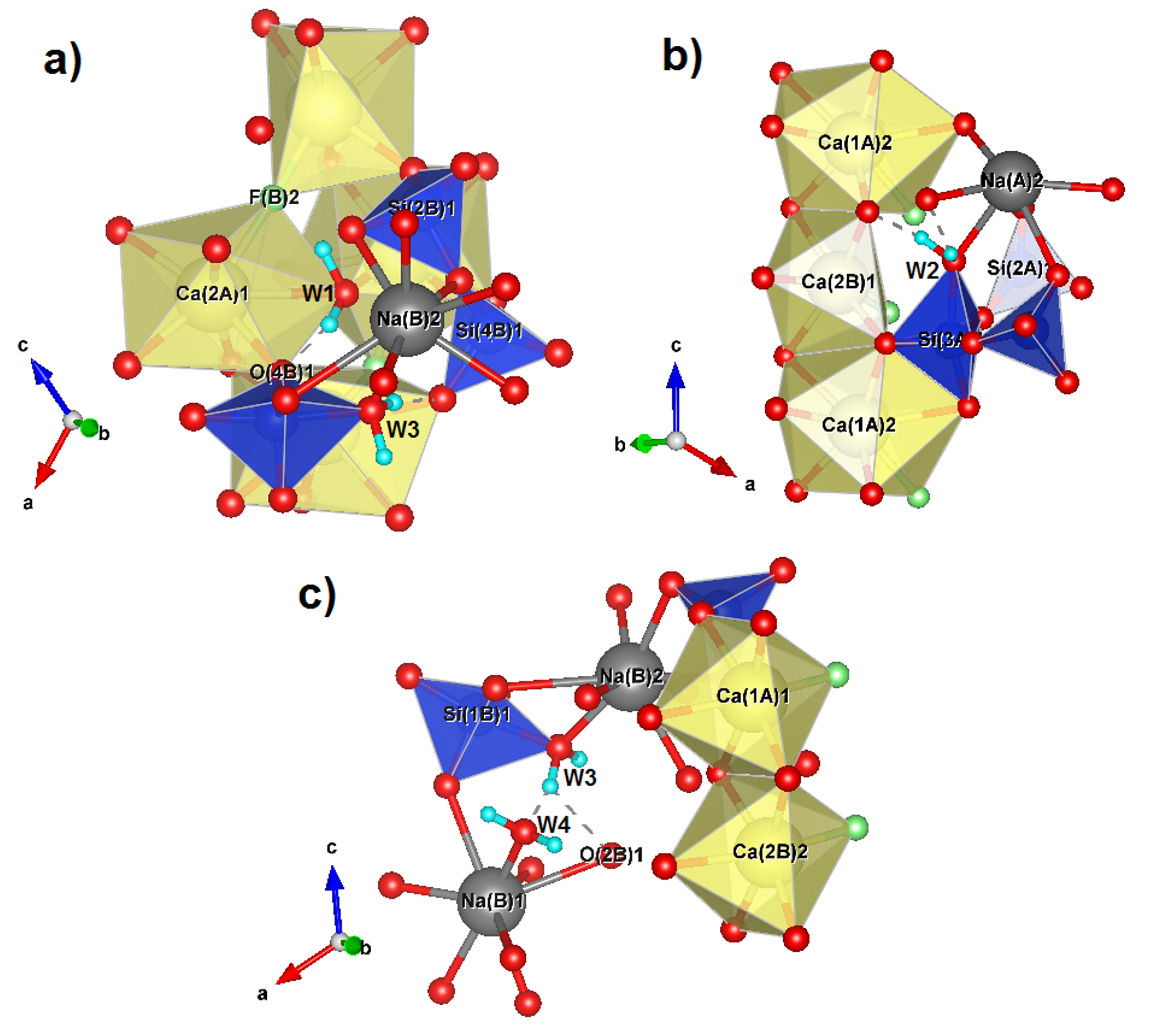
**

**Figure S5**. Positions of the H_2_O molecules in the simulated structural model C3 with four H_2_O molecules per unit cell (4.4 wt.%): *a*) W1 and W3 molecules in the p1 and p3 positions, *b*) W2 molecule in the p2 position, *c*) W3 and W4 molecules in the p3 and p4 positions. The definitions of colors are the ASme as in Figure S3.

**
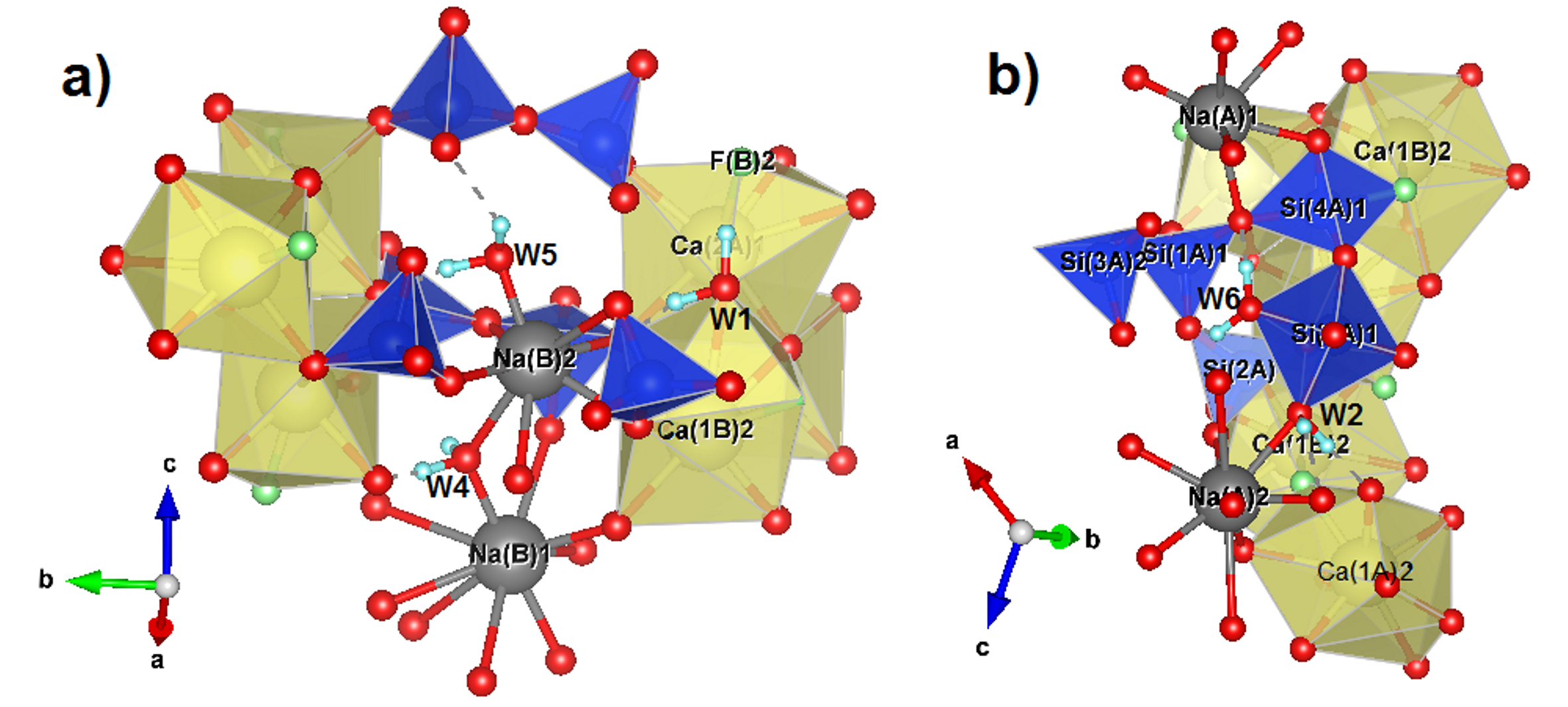
**

**Figure S6**. Positions of the H_2_O molecules in the simulated structural model C4 with five H_2_O molecules per unit cell (5.3 wt.%): *a*) W1, W4 and W5 molecules in the p1, p4 and p5 positions, respectively, *b*) W2 and W6 molecules in the p2 and p6 positions. The definitions of colors are the ASme as in Figure S3.

**
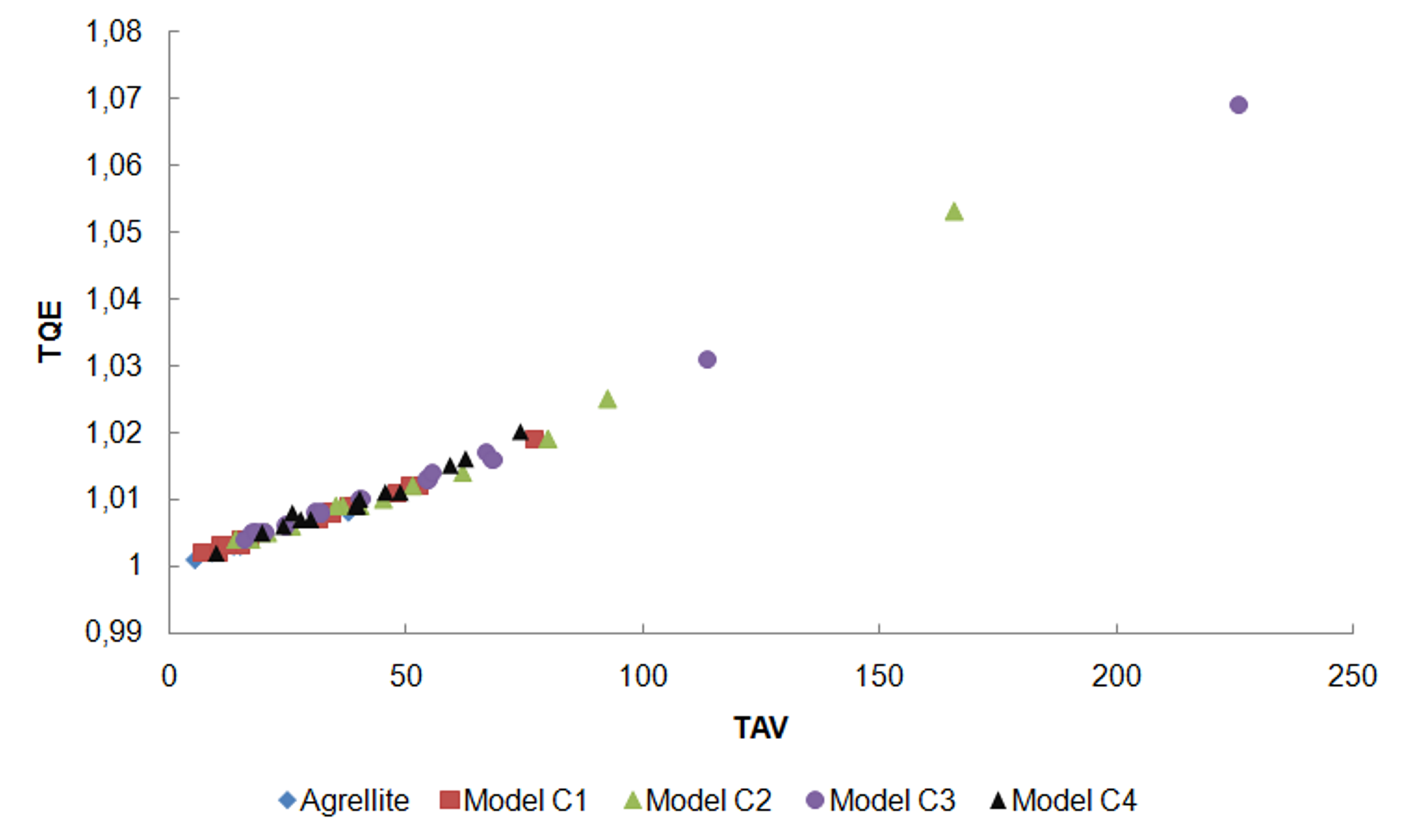
**

**Figure S7**. Mean quadratic elongation for tetrahedra in the ideal model and models C1-C4 of agrellite crystal structure against tetrahedral angle variance.

**
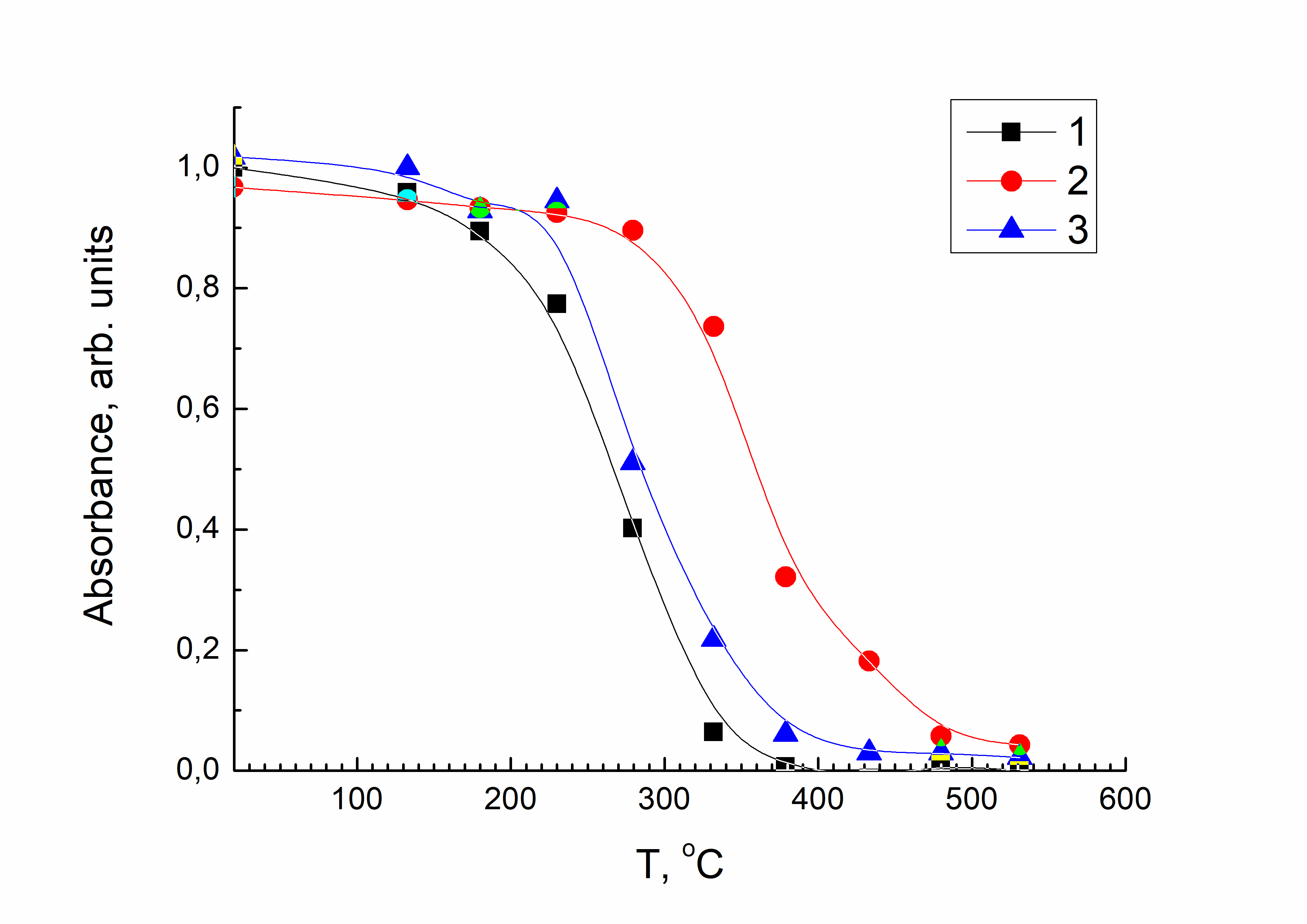
**

**Figure S8**. Integral absorbance of peaks at 3065 (curve 1), 3470 (curve 2), 3555 cm^-^1 (curve 3) on agrellite preheating temperature.
